# Myosin VI regulates the spatial organisation of mammalian transcription initiation

**DOI:** 10.1038/s41467-022-28962-w

**Published:** 2022-03-15

**Authors:** Yukti Hari-Gupta, Natalia Fili, Ália dos Santos, Alexander W. Cook, Rosemarie E. Gough, Hannah C. W. Reed, Lin Wang, Jesse Aaron, Tomas Venit, Eric Wait, Andreas Grosse-Berkenbusch, J. Christof M. Gebhardt, Piergiorgio Percipalle, Teng-Leong Chew, Marisa Martin-Fernandez, Christopher P. Toseland

**Affiliations:** 1 School of Biosciences, University of Kent, Canterbury, UK; 2 Department of Oncology and Metabolism, University of Sheffield, Sheffield, UK; 3 Central Laser Facility, Research Complex at Harwell, Science and Technology Facilities Council, Rutherford Appleton Laboratory, Harwell, Didcot, Oxford, UK; 4 Advanced Imaging Center, HHMI Janelia Research Campus, Ashburn, VA USA; 5 Science Division, Biology Program, New York University Abu Dhabi (NYUAD), Abu Dhabi, United Arab Emirates; 6 Institute of Biophysics, Ulm University, Ulm, Germany; 7 Department of Molecular Bioscience, The Wenner Gren Institute, Stockholm University, Stockholm, SE Sweden; 8 Present Address: MRC LMCB, University College London, London, UK; 9 Present Address: School of Life Sciences, University of Lincoln, Lincoln, UK

**Keywords:** DNA, Imaging, Nanoscale biophysics, Super-resolution microscopy, Nuclear organization

## Abstract

During transcription, RNA Polymerase II (RNAPII) is spatially organised within the nucleus into clusters that correlate with transcription activity. While this is a hallmark of genome regulation in mammalian cells, the mechanisms concerning the assembly, organisation and stability remain unknown. Here, we have used combination of single molecule imaging and genomic approaches to explore the role of nuclear myosin VI (MVI) in the nanoscale organisation of RNAPII. We reveal that MVI in the nucleus acts as the molecular anchor that holds RNAPII in high density clusters. Perturbation of MVI leads to the disruption of RNAPII localisation, chromatin organisation and subsequently a decrease in gene expression. Overall, we uncover the fundamental role of MVI in the spatial regulation of gene expression.

## Abstract (web summary)

The actin-based molecular motors, myosins, have also been linked to transcription, but their precise role has remained elusive. Here the authors show RNA polymerase II (RNAPII) is lost from chromatin upon myosin perturbation and that myosin acts as a molecular anchor to maintain RNAPII spatial organisation.

## Introduction

**Fig. 1:**
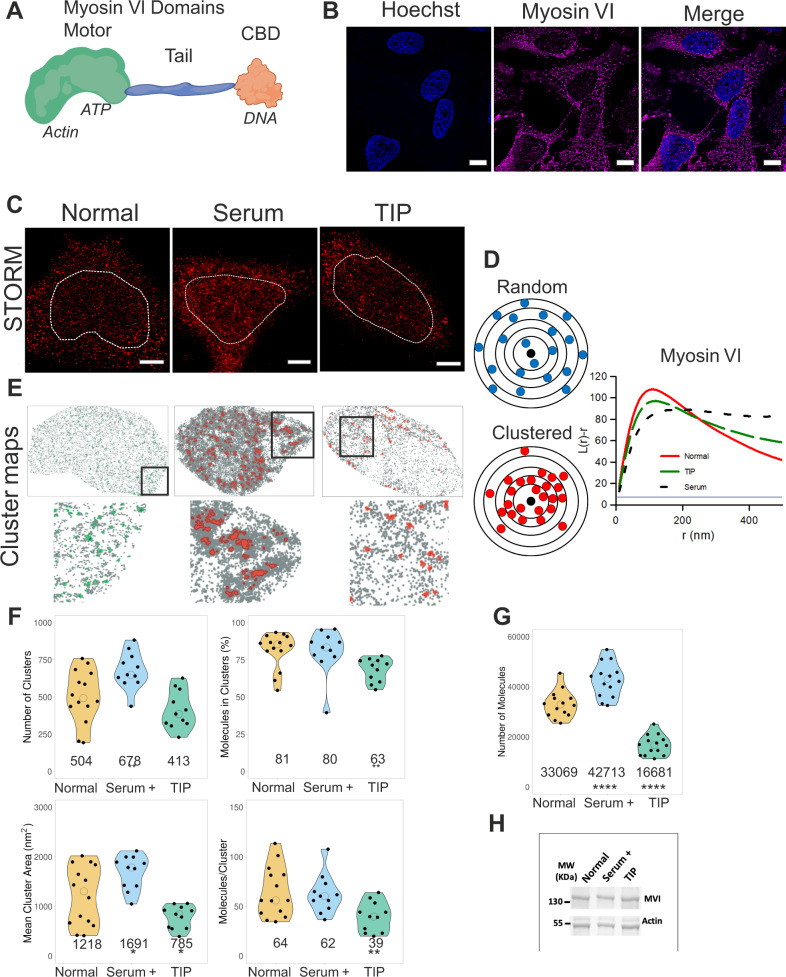
Nuclear organisation of myosin VI. **A** Cartoon depiction of the MVI key features including the cargo-binding domain (CBD). **B** Widefield Immunofluorescence staining against MVI (magenta) and DNA (cyan) in HeLa cells. Images were acquired at the mid-point of the nucleus (Scale bar 10 μm). (See Supplementary Figs. 1, 2 for confocal z stack and antibody staining control). **C** Example STORM render images of MVI under normal, serum+ and TIP-treated conditions (scale bar 2 μm). Dotted lines represent a region of interest (ROI) containing the nucleus which are taken forward for cluster analysis. The nucleus was identified using either Hoechst or RNAPII staining. **D** Depiction of molecular clustering and random distribution. We performed cluster analysis using the linearised form of Ripley’s K function^21^ *L(r)-r*, where r is the radius. A plot of *L(r)-r* versus *r* gives a value of zero for a random distribution (blue line), but deviates from zero, towards positive values, due to molecular clustering (red). The organisation of MVI is seen with peaks at 125 ± 45 nm (normal), 120 ± 25 nm (TIP) and 200 ± 30 nm (Serum). **E** Cluster maps based upon the selected ROI in (**C**). Clusters are shown in green (Normal) or red (Serum and TIP treatment). Selected regions are shown at higher magnification. **F** Cluster analysis of MVI nuclear organisation under normal, serum+ and TIP-treated conditions. Individual data points correspond to the average value for a cell ROI (*n* = 14 for normal, 11 for serum+ and TIP-treated). The values represent the mean from the ROIs for each condition (Only statistically significant changes are highlighted *p < 0.05; **p < 0.01 by two-tailed *t* test compared to normal conditions). **G** Number of localisations of MVI under the different conditions. Individual data points correspond to the value for a cell ROI (*n* = 14). The values represent the mean from the ROIs for each condition (****p < 0.0001 by two-tailed *t* test compared to normal conditions). **H** Western-blot against MVI under Normal, Serum and TIP-treated conditions. The expression levels relative to normal are 0.95 and 1.05 for serum and TIP-treated conditions, respectively.

The tight regulation of gene expression is critical for the maintenance of cellular homoeostasis. In eukaryotic cells, RNAPII directs the flow of genetic information from DNA to messenger RNA. Detailed genetic and biochemical assays have revealed a multi-level regulation of transcription, including *cis* control elements and *trans* factors. The actin-based molecular motors, myosins, have been also shown to act as transcription regulators^1–4^ in addition to their multiple cytoplasmic functions^5^. MVI (Fig. 1A), has been shown to bind DNA through its cargo-binding domain (CBD) and couple itself to RNAPII in an actin-dependent manner through the motor domain^6^. It has been revealed that the ability of MVI to bind DNA and its ATPase activity are both critical for transcription in vitro^6–8^. Recently, MVI has been shown to actively undergo directed motion in the nucleus in response to transcription stimulation^9^. However, the precise role that this motor protein has in transcription has remained elusive.

The spatial organisation of transcription has been studied by both imaging and immunoprecipitation methods for over two decades^10–12^. The formation of transcription centres, termed transcription factories or condensates^11,13^, has been suggested to increase the local concentration of enzymes and render these nuclear processes more efficient^14^. The lifetime and composition of these clusters has been a matter of debate^11,12,15–18^. More recently, RNAPII has been found to transiently cluster during transcription^10,19^. Yet, detailed molecular mechanisms of how these clusters form and how they are maintained remain unknown.

Interestingly, the biochemical properties of MVI can be tuned by the load applied to the motor^20^, which allows it to switch from an active transporter to an actin anchor when tension is applied. We therefore hypothesised that MVI could act as either an anchor to stabilise RNAPII, or as an auxiliary motor to drive RNAPII. In either case, this would impact the organisation of RNAPII within the nucleus.

In this work, we show how MVI activity contributes to the spatial organisation of RNAPII. We have endeavoured to provide general mechanistic insight into how this form of nuclear organisation is achieved and what is the role of a myosin in this process.

## Results

### The nuclear organisation of myosin VI

MVI is present throughout the mammalian cell, including the nucleus (Figs. 1B, S1 and Movie S1). To gain better understanding of the spatial organisation of MVI, we used super resolution imaging, specifically Stochastic Optical Reconstruction Microscopy (STORM) (Fig. 1C and Supplementary Fig. 3). This approach was combined with illuminating the sample with a Highly Inclined and Laminated Optical sheet (HILO) to specifically excite a narrow 1 µm band through the nucleus while excluding the cytoplasm above and below. This enabled individual MVI molecules within the nucleus to be resolved, quantified and their functional clustering behaviour assessed. To determine whether MVI assembles into clusters or is randomly distributed, we performed cluster analysis using the linearised form of Ripley’s K function^21^ (Fig. 1D). This analysis demonstrated that nuclear myosin VI is clustered, rather than randomly distributed. To further understand this clustering behaviour, we used the Clus-DoC software^21^, which allows to quantify the spatial distribution of a protein using DBSCAN leading to the detection of clusters and generation cluster maps (Fig. 1E). Clusters are defined by detecting a minimum of three molecules within a search area corresponding to the STORM localisation precision. The search area then propagates and a group of molecules is considered to be a cluster if at least 10 molecules are found. We were able to determine that 81% of MVI is clustered, with an average of 504 clusters per nuclei. Each cluster, with an average cluster size of 1200 nm^2^, consists of 64 MVI molecules (Fig. 1F).

Given the well-established role of MVI in transcription^6–9,22^, we assessed whether stimulation of transcription can alter its clustering and thus functional properties. Serum stimulation activates transcription of specific serum-responsive genes which regulate the SRF-MAL pathway^23,24^. Treatment with serum induced a significant increase in the nuclear distribution of MVI, as evidenced by both the STORM imaging and quantification (Fig. 1C, G and Supplementary Fig. 3). Importantly, the increase in number of molecules was specific to a nuclear recruitment because the global level of protein was unchanged (Fig. 1H). Consistent with the noticeable increase in its nuclear recruitment, the number of clusters and their area increased significantly to 678 clusters per nuclei and 1700 nm^2^, respectively (Fig. 1F). Consistent with this observation, serum starvation led to a significant decrease in the number and size of the clusters (Supplementary Fig. 4).

Since MVI is an ATPase, we then explored whether its nuclear distribution and clustering behaviour was dependent upon its myosin motor activity. To this end, we used the small-molecule inhibitor (TIP) which is known to perturb the motor activity of MVI^25^ and impact upon transcription^6–8^. Overall, there was a significant decrease in the nuclear fraction of MVI by half (Fig. 1G) but no change in total protein (Fig. 1H). This disrupted the nuclear organisation of MVI by decreasing the number of molecules in a cluster (Fig. 1C, E, F and Supplementary Fig. 3) suggesting motor activity is required for nuclear recruitment and organisation.

Under normal conditions, there is a large variance in the MVI clustering data and potentially two populations, especially in the cluster size. The serum stimulation and TIP experiments suggest that these could be active and inactive populations. However, it may also relate to distinct populations of MVI engaged in different roles.

**Fig. 2:**
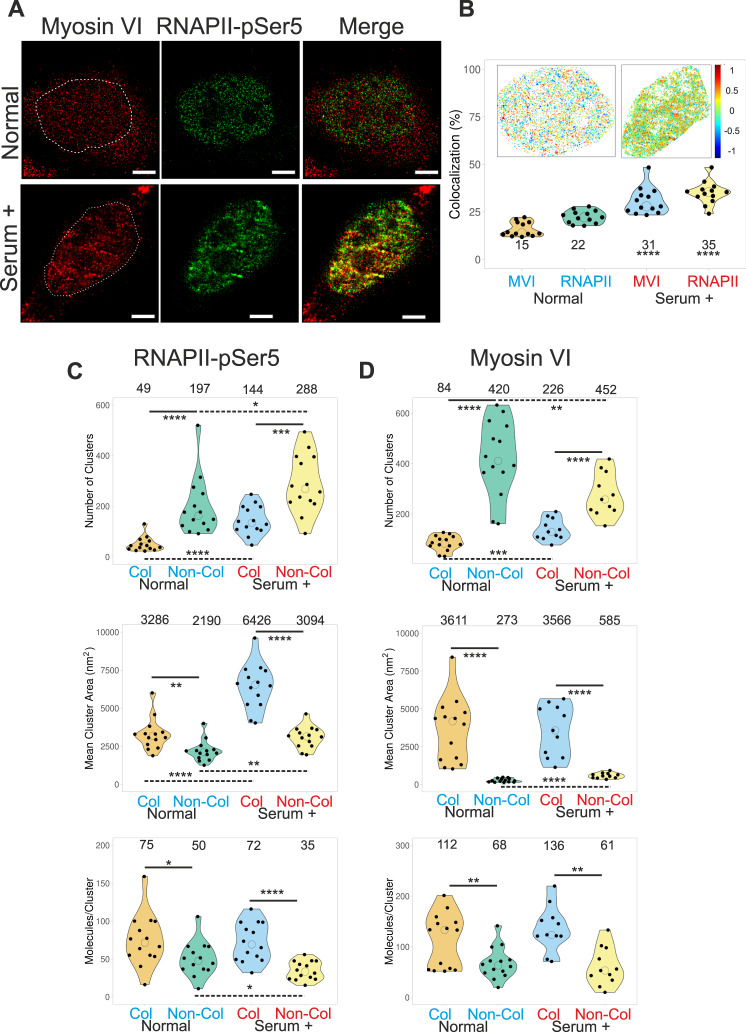
Nuclear organisation of RNAPII and colocalization with Myosin VI. **A** Example STORM render image of MVI and RNAPII-pSer5 under normal conditions and serum-treated conditions (scale bar 2 μm). **B** Co-localisation analysis of MVI and RNAPII-pSer5 clusters under normal and serum-treated conditions. Inset is a representative cluster colocalization heatmap whereby DoC values of 1 are perfectly colocalised and −1 are separated from each other. Individual data points represent the percentage of each protein which is colocalized based on DoC threshold of above 0.4 (*n* = 13) (see Fig. S6 for example histograms). The values represent the mean from the ROIs for each protein (Only statistically significant changes are highlighted ****p < 0.0001 by two-tailed *t* test compared to normal conditions for each protein). **C** Cluster analysis of RNAPII-pSer5 nuclear organisation under normal and serum-treated conditions. The data are broken down into myosin VI colocalized and non-colocalized clusters. Individual data points correspond to the average value for a cell ROI (*n* = 14). The values represent the mean from the ROIs for each condition (Only statistically significant changes are highlighted *p < 0.05; **p < 0.01; ***p < 0.001; ****p < 0.0001 by two-tailed *t* test compared to normal conditions). **D** Cluster analysis of myosin VI nuclear organisation under normal and serum-treated conditions. The data are broken down in to RNAPII-pSer5 colocalized and non-colocalized clusters. Individual data points correspond to the average value for a cell ROI (*n* = 14). The values represent the mean from the ROIs for each condition (Only statistically significant changes are highlighted **p < 0.01; ***p < 0.001; ****p < 0.0001 by two-tailed *t* test compared to normal conditions).

Having observed that transcription stimulation drives MVI cluster formation, we turned our attention to RNAPII to explore if there is a relationship between the clustering of both proteins. Firstly, we imaged RNAPII in the transcription initiation state (pSer5) using STORM and performed cluster analysis (Fig. 2A and Supplementary Fig. 5A). As it has previously been shown^10,11^, we also observed clusters of RNAPII under normal growth conditions, whereby 42% of RNAPII was clustered, into 246 clusters per cell, each containing 60 molecules in an area of 2700 nm^2^, on average (Supplementary Fig. 5A). Interestingly, these clusters partially colocalized with the MVI clusters (Fig. 2A). In order to quantify this colocalization, we employed the Degree of Co-localisation (DoC) analysis^21^ which employs a coordinate-based method to determine correlation between individual molecules in each channel^26^. A histogram of DoC scores for each channel is presented in Fig. S6. The colour-coded co-localisation cluster map reflects the DoC value for each molecule. Applying a threshold of 0.4 allows us to determine that 15% of MVI and 22% of RNAPII molecules colocalise (Fig. 2B). The single-molecule nature of these measurements allowed us to further interrogate the data by comparing the features of clusters consisting of colocalised and non-colocalised molecules. Whilst the number of colocalised clusters for both proteins is lower than the non-colocalised ones, these clusters are up to 1.5-fold larger in size for RNAPII and up to 10-fold larger for MVI, compared to the non-colocalised subpopulation (Fig. 2C, D). This also correlates with an approximate 50% and 100% increase in RNAPII and MVI molecules, respectively, within the colocalised clusters. Overall, this suggests there is synergy between populations of the two proteins. Nevertheless, there are significant pools of protein acting independently.

To further understand the functional relevance of the MVI-RNAPII co-localisation, we stimulated transcription using serum (Fig. 2A). Similar to MVI, there was a noticeable change in the RNAPII distribution, consistent with previous observations^27,28^. Serum stimulation led to an increase in cluster size and number of clusters of RNAPII (Supplementary Fig. 5A). Moreover, we observed a significant increase in positive colocalization between the clusters, where 31% and 35% of MVI and RNAPII colocalized, respectively (Fig. 2B). Furthermore, comparison of colocalised and non-colocalised subpopulations showed that the synergy between the two proteins is maintained following serum stimulation (Fig. 2C). Consistent with these measurements, serum starvation decreased cluster formation and colocalization (Supplementary Fig. 5).

### Myosin VI regulates the spatial organisation of RNA Polymerase II

After observing the correlation between RNAPII and MVI clustering, and building upon the established role of MVI in transcription, we wanted to explore how MVI activity may impact the spatial organisation of RNAPII which underpins mammalian gene expression.

**Fig. 3:**
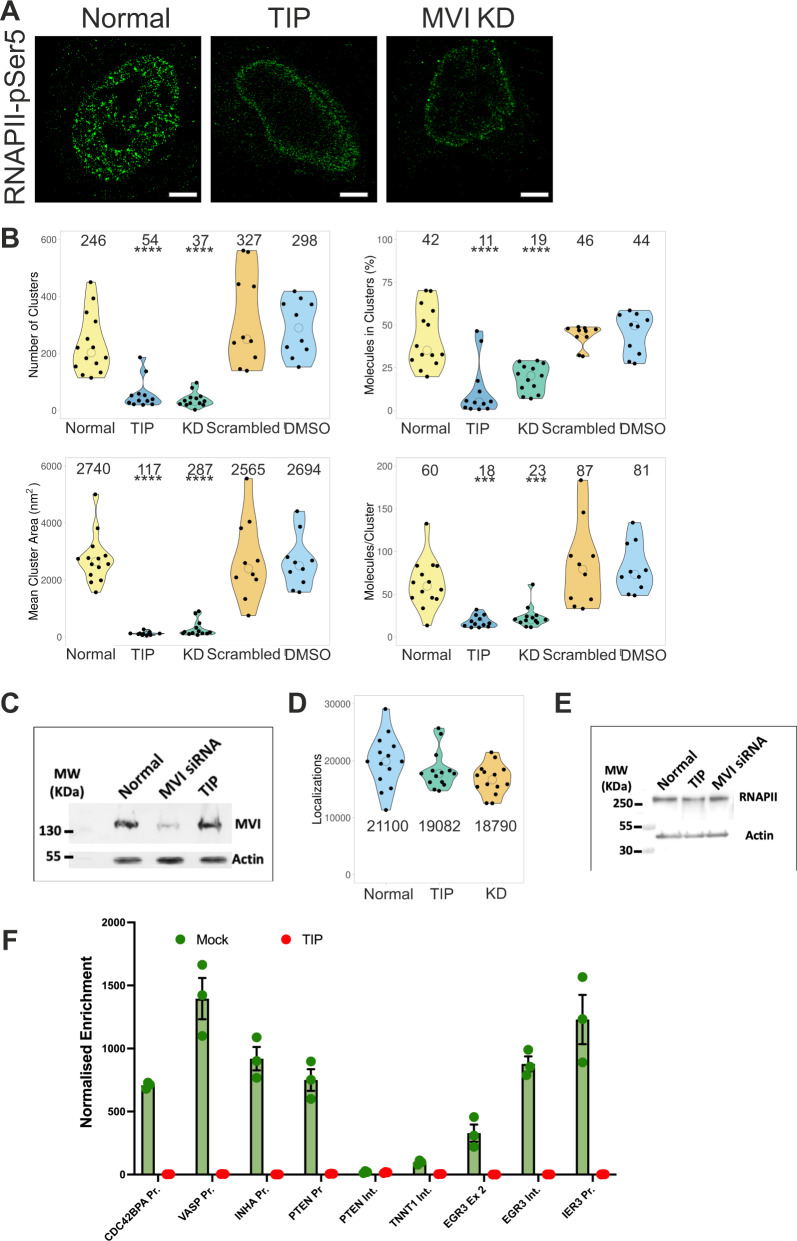
Spatial organisation of RNAPII depends upon myosin VI. **A** Example STORM render image of RNAPII-pSer5 under normal, TIP-treated and MVI (MVI) knockdown conditions (scale bar 2 μm). Further widefield example images are shown in Supplementary Figs. 7, 10. **B** Cluster analysis of RNAPII nuclear organisation under the conditions described in (**A**). Individual data points correspond to the average value for a cell ROI (*n* = 14 for normal, 12 for TIP, 13 for KD, 10 for Control siRNA and DMSO). The values represent the mean from the ROIs for each condition (Only statistically significant changes are highlighted ***p < 0.001; ****p < 0.0001 by two-tailed *t* test compared to normal conditions). **C** Western-blot against MVI under Normal, MVI-knockdown (MVI siRNA) and TIP-treated conditions. Quantification relative to Normal shows a 95% reduction for MVI siRNA. TIP represents 103% relative to Normal. Example widefield images of MVI under knockdown conditions are shown in Supplementary Fig. 2. **D** Number of localisations of RNAPII-pSer5 under the different conditions. Individual data points correspond to the value for a cell ROI (*n* = 14). The values represent the mean from the ROIs for each condition. **E** Western-blot against RNAPII-pSer5 under normal, TIP-treated and MVI-knockdown (MVI siRNA) conditions. Quantification relative to Normal shows a 5% reduction for TIP. MVI siRNA represents 105% relative to Normal. **F** RNAPII-pSer5 ChIP against labelled loci under normal and TIP-treated conditions. Values are the average of three independent experiments. Error bars represent SEM from three independent experiments.

Treatment with the MVI inhibitor TIP induced a significant disruption of the spatial distribution of RNAPII, whereby the protein was aggregated at the nuclear periphery, while it was significantly decreased in the centre of the nucleus (Fig. 3A and Supplementary Fig. 7). Not surprisingly, based on the visual redistribution of RNAPII, cluster parameters were significantly decreased, compared to normal conditions (Fig. 3B). We also confirmed that, while TIP impacts the clustering activity of MVI (Fig. 1C), it does not lead to protein degradation (Fig. 3C), changes in the cytoskeleton (Supplementary Fig. 8A), noticeable changes in the cell shape (Supplementary Fig. 8B), or change to nuclear size and integrity (Supplementary Fig. 9), consistent with previous observations^25^. We therefore conclude that the motor activity of MVI is required for RNAPII clustering. To further support this finding, we performed siRNA transient knockdown of MVI (Fig. 3A–C, Supplementary Figs. 2, 10). Similar to the effect of the inhibitor, absence of MVI had a significant impact on the RNAPII distribution.

Based on the number of localisations in the STORM imaging and western-blot analysis, we confirmed that the total amount of RNAPII-pSer5 did not change following either treatment (Fig. 3D, E). Therefore, perturbation of MVI causes destabilisation of RNAPII clusters. Consistently, TIP treatment also led to a decrease in MVI-RNAPII colocalization (Supplementary Fig. 5C). Overall, our data indicated a role for MVI in the nuclear organisation of RNAPII.

We next sought to establish whether the redistribution of RNAPII also correlated with its loss from chromatin. To this end, Chromatin Immunoprecipitation (ChIP) against RNAPII-pSer5 was performed under normal conditions and following TIP treatment. Indeed, MVI inhibition induced a decrease of several orders of magnitude in RNAPII occupancy from a selection of serum-responsive genes (Fig. 3F and Supplementary Fig. 11).

Both MVI knockdown and inhibition by TIP have an impact on the cytoplasmic and nuclear populations of MVI. To determine the specific role of the nuclear population of MVI in the spatial organisation of RNAPII, we transfected cells with NLS-tagged truncations of MVI, namely NLS-CBD (Cargo-binding domain) and NLS-Motor. Based on in vitro transcription assays^6^, over-expression of these constructs and their targeting to the nucleus was expected to have a dominant-negative impact upon the endogenous nuclear MVI by displacing the protein, as occurs in the cytoplasm^29–32^. Indeed, over-expression of either construct disrupted the nuclear distribution of RNAPII, as observed by widefield microscopy (Supplementary Fig. 12A, B). Moreover, constructs confide to the cytoplasm, which compete with cytoplasmic myosin VI^29^, did not impact RNAPII organisation (Supplementary Fig. 12C, D). We therefore concluded that it is the nuclear pool of MVI that is directly involved in the nuclear organisation of RNAPII.

**Fig. 4:**
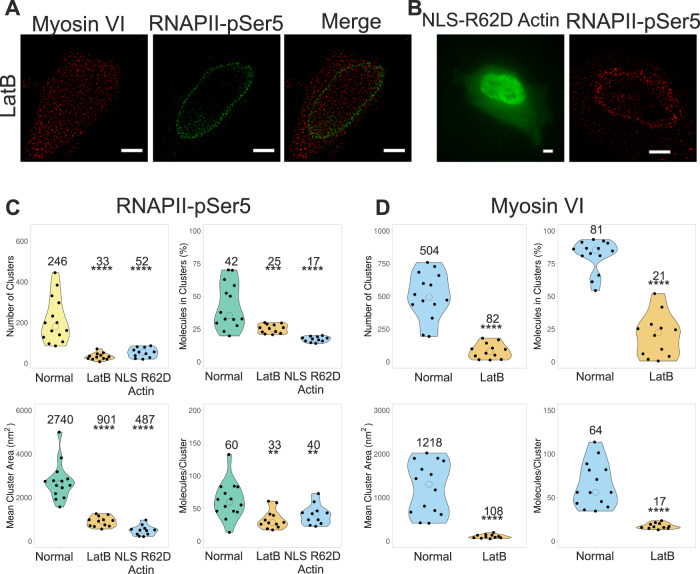
Impact of nuclear actin upon the organisation of RNAPII. **A** Example STORM render image of myosin VI and RNAPII-pSer5 following treatment with Latrunculin B (LatB), as described in the methods (scale bar 2 μm). Further widefield example images are shown in Supplementary Fig. 13D. **B** (left) Example widefield image of YFP-NLS-R62D actin following transfection. (right) Example STORM render image of RNAPII-pSer5 following transfection of YFP-NLS-R62D actin (scale bar 2 μm) WT YFP-NLS actin control is Supplementary Fig. 13E. **C** Cluster analysis of RNAPII-pSer5 nuclear organisation following treatment with LatB (*n* = 12) or transfection with YFP-NLS-R62D Actin (*n* = 11). **D** Cluster analysis of myosin VI nuclear organisation following treatment with LatB (*n* = 12). Individual data points correspond to the average value for a cell ROI. The values represent the mean from the ROIs for each condition (Only statistically significant changes are highlighted **p < 0.01; ***p < 0.001; ****p < 0.0001 by two-tailed *t* test compared to normal conditions).

We next explored whether the impact of MVI on RNAPII is also dependent upon nuclear actin. It has been well-established that actin is bound to RNAPII^1,2,33^ and nuclear actin was recently found to support clustering of RNAPII^34^. Consistent with this previous report, we also observed clusters of actin within the nucleus (Supplementary Fig. 13A) with dense regions of actin are seen closer to the nuclear periphery^34^. We have been able to colocalize MVI clusters with actin (Supplementary Fig. 13B, C) but due to both motor and CBD-dependent interactions within the nucleus, clusters of MVI exist without actin. We have not been able to performed three colour STORM experiments to observe MVI, RNAPII and actin together due to technical limitations. We subsequently performed two types of actin perturbation experiments: (a) Treatment with latrunculin B to prevent actin polymerisation, which would reveal whether actin polymers are important for transcription. (b) Transient expression of a nuclear targeted monomeric actin mutant, namely NLS-YFP-R62D-actin^35^. This mutant would outcompete endogenous nuclear G-actin, thereby preventing its polymerisation into filaments. Both of these perturbations caused disruption of RNAPII organisation (Fig. 4A, B) but over-expression of NLS-WT-actin did not (Supplementary Fig. 13E), which supports the work shown recently^34^. Cluster analysis (Fig. 4C) shows that all cluster parameters are significantly decreased in both conditions, as is the colocalization between MVI and RNAPII with latrunculin B (Supplementary Fig. 5C). These results were also supported by the impact of latrunculin B treatment on the nuclear organisation of MVI. Similar to RNAPII, all clustering parameters for MVI were significantly decreased (Fig. 4D), leading to a greater impact than TIP treatment (Fig. 1). This suggests, that the nuclear roles of MVI involve its interaction with filaments or short polymers of actin. Overall, these results indicate that the MVI-actin interaction is required for the correct spatial organisation of RNAPII.

### Myosin VI controls the nuclear dynamics of RNA Polymerase II

Having revealed the role of MVI in the nuclear organisation of RNAPII, we then assessed its role in the dynamics of RNAPII in living cells to understand the assembly of the transcription factories.

**Fig. 5:**
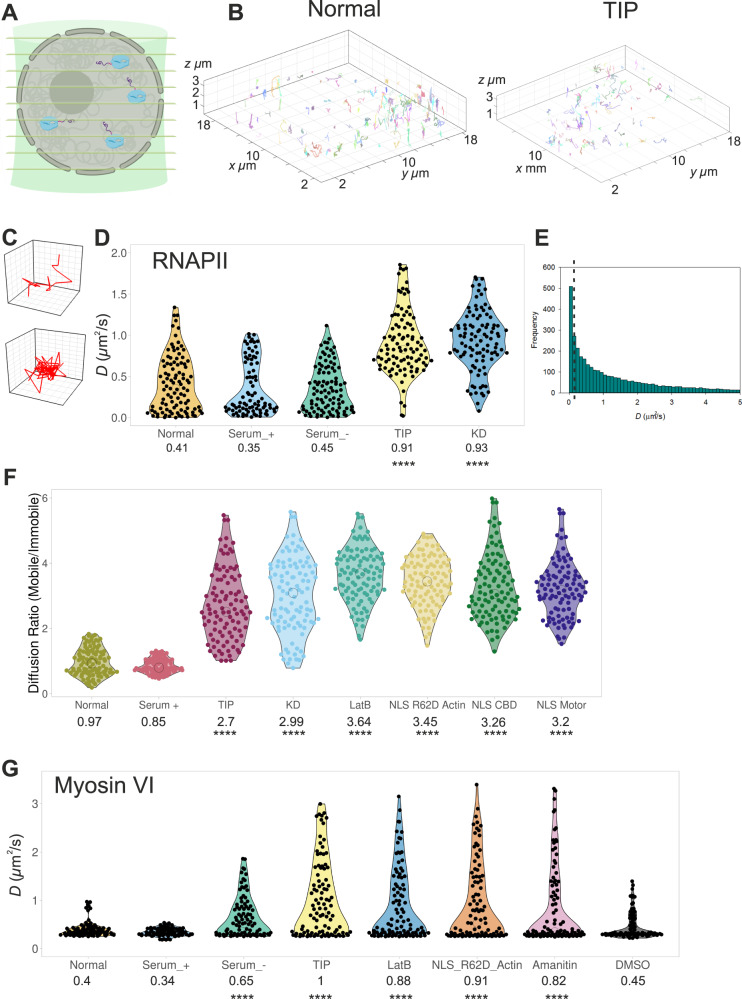
Live-cell single-molecule dynamics of RNAPII. **A** Cartoon depicting simultaneous acquisition of 9 focal planes covering 4 μm to perform live-cell 3D single-molecule tracking of RNAPII. **B** Example render of 3D single-molecule trajectories under normal and TIP-treated conditions. Further examples are shown in Supplementary Fig. 15. **C** Example trajectory of a diffusive and spatially confined molecule which can be identified in (**B**). **D** Plot of Halo-RNAPII diffusion constants under the stated conditions derived from fitting trajectories to an anomalous diffusion model, as described in methods. Individual data points correspond to the average value for a cell ROI (*n* = 100). The values represent the mean from the ROIs for each condition (Only statistically significant changes are highlighted ****p < 0.0001 by two-tailed *t* test compared to normal conditions). **E** Example histogram of diffusion constants arising from a single cell. The dotted line represents the threshold applied to segregate static and dynamic molecules. **F** Using the threshold defined in (**E**), trajectories were plotted as a ratio of mobile and immobile species. Individual data points correspond to the average value for a cell ROI. The values represent the mean from the ROIs for each condition (Only statistically significant changes are highlighted ****p < 0.0001 by two-tailed *t* test compared to normal conditions). NLS-R62D Actin, NLS CBD and NLS Motor refer to tracking of RNAPII following transfection of these constructs. NLS CBD and NLS motor were transfected in to Halo-RNAPII cells. **G** Plot of Halo-MVI diffusion constants under the stated conditions derived from fitting trajectories to an anomalous diffusion model, as described in methods. Individual data points correspond to the average value for a cell ROI (*n* = 100). The values represent the mean from the ROIs for each condition (Only statistically significant changes are highlighted ****p < 0.0001 by two-tailed *t* test compared to normal conditions).

To achieve this, we performed single-molecule tracking of Halo-tagged or SNAP-tagged Rbp1, the largest RNAPII subunit, using an aberration-corrected multifocal microscope (acMFM) system^36^ (Fig. 5A). In this way, we were able to observe and track the 3D dynamics of RNAPII across the whole nucleus, in live cells (Fig. 5B and Supplementary Movies 2, 3). Clustering of RNAPII was not observed in these live-cell experiments due to the low labelling density required in order to achieve single-molecule detection in the crowded nuclear environment. Moreover, all populations of RNAPII were visible, compared to solely the pSer5 population in the STORM measurements. We observed pools of spatially confined RNAPII molecules and pools of molecules diffusing freely within the nucleus (Fig. 5C). We determined the diffusion constant for each track by measuring the Mean Squared Displacement (MSD) and then plotted the average diffusion constant per cell (Fig. 5D and Supplementary Fig. 14A). Under normal conditions, we found that, on average, RNAPII diffuses relatively slowly (0.41 µm^2^ s^−1^) and this decreases further during transcription stimulation (0.35 µm^2^ s^−1^). When we interrogate the individual tracks for each cell, we observed several populations of RNAPII which we termed as (i) static *D* < 0.1 µm^2^ s^−1^, (ii) diffusive 0.1 < D < 5 µm^2^ s^−1^ and (iii) hyper mobile (not quantified) (Fig. 5E). These findings are consistent with the previous reports using this technique^36^. To further investigate RNAPII dynamics, the total numbers of static (<0.1 µm^2^ s^−1^) and mobile (>0.1 µm^2^ s^−1^) RNAPII molecules were plotted as a ratio (Fig. 5F). Under normal conditions, just over half of the RNAPII population (52%) was static, probably corresponding to molecules confined at sites of transcription activation.

Similar to the STORM experiments, we then observed the dynamics of RNAPII following TIP treatment, siRNA knockdown of MVI and actin perturbations. These treatments led to a twofold increase in the RNAPII average diffusion constant (Fig. 5D and Supplementary Fig. 14A) and three to fourfold increase in the motile fraction (Fig. 5F). Visually, the impact was also clear and could be observed through the loss of spatially confined molecules and the gain of diffusive tracks (Fig. 5B). This was further quantified by plotting the anomalous diffusion alpha values whereby TIP treatment leads to an increase in freely diffusing species (Supplementary Fig. 14B). In order to assess whether TIP has a global impact on molecular diffusion in the nucleus, we transiently expressed an isolated SNAP-tag domain to act as a diffusion reporter for the nuclear environment (Supplementary Fig. 14C). We would not expect any impact on the diffusion of this isolated protein domain when cells are treated with TIP. Indeed, no changes were observed, confirming that the detected changes in RNAPII behaviour relate solely to the activity of MVI. We also observed the RNAPII dynamics in cells transiently expressing the dominant-negative NLS-motor and NLS-CBD constructs. Consistent with the effect of TIP and MVI knockdown, there was an almost twofold increase in RNAPII diffusion in both cases, as well as an increase in the motile fraction of RNAPII (Fig. 5F and Supplementary Fig. 14A). This increased mobility of RNAPII following perturbation of MVI would be expected to lead to a decrease in the number of clusters, which is what we observed with the STORM measurements. Moreover, the greater mobility of RNAPII would also account for its relocation to the nuclear periphery, where it may non-specifically associate with the nuclear membrane or lamina. Overall, our observations suggest a model whereby MVI stabilises the RNAPII at sites of transcription initiation.

Finally, we also explored the nuclear dynamics of MVI and its interplay with RNAPII (Fig. 5G). Overall, MVI is relatively static, with a mean diffusion constant of 0.4 µm^2^ s^−1^. However, treatment with TIP, or perturbation of actin, resulted in an increased mean diffusion to ~1 µm^2^ s^−1^, which is consistent with the STORM measurements, that show a reduction in clustering behaviour. Moreover, serum starvation increase diffusion to 0.65 µm^2^ s^−1^ suggesting transcription activity is linked to MVI organisation. Interestingly, a twofold increase in mean MVI diffusion was observed when cells were treated with the RNAPII inhibitor α-amanitin, that inhibits transcription through RNAPII degradation (Fig. 5G and Supplementary Fig. 14D–F). Altogether, we propose that loss of RNAPII reduces MVI binding events to RNAPII therefore increasing diffusion.

### RNA Polymerase II spatial distribution is coupled to transcription activity

**Fig. 6:**
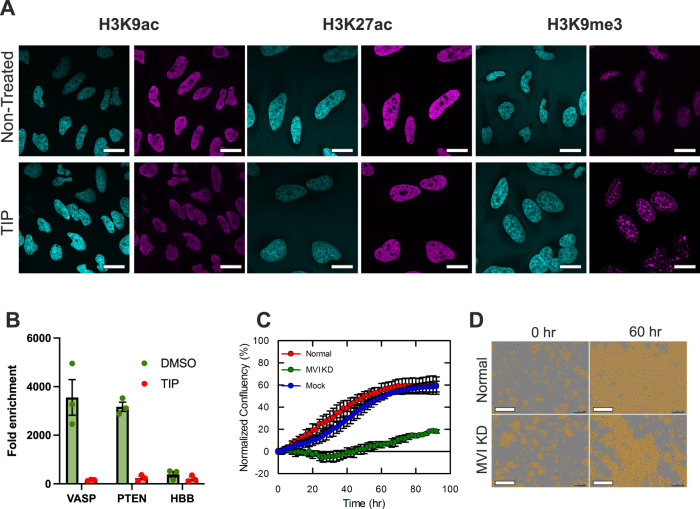
Perturbation of myosin VI impacts chromatin organisation and cell growth. **A** Example immunofluorescence staining against stated histone marks (magenta) and DNA (blue) in HeLa cells under normal and TIP-treated conditions (scale bar 10 μm). **B** Chromatin accessibility assay. qPCR was performed against the stated genes on chromatin purified from HeLa cells in the presence and absence of TIP. Chromatin was divided in to nuclease and control samples where nuclease activity was determined by difference between the two samples (Fold enrichment). *HBB* represents a repressed, inaccessible, control gene. Error bars represent SEM from three independent experiments. **C** Real-time growth of HeLa cells (red) and corresponding measurements following MVI (MVI) siRNA knockdown (green) and mock transfection control (blue). Data represent three independent measurements and error bars show SEM. Example images at start and 60 h time points are shown in (**D**) (scale bar 300 μm in all images). Western blots are shown in Supplementary Fig. 17 for the MVI knockdown.

We then explored the impact of the perturbed nuclear organisation of RNAPII on the underlying chromatin which could fundamentally alter the cellular properties. We used antibodies against histones H3K9ac and H3K27ac, positive epigenetic marks of active gene expression, and H3K9me3, a mark of repressed transcription^37^. We performed imaging under normal growth conditions and TIP treatment. H3K9ac and H3K27ac showed a decrease in staining following TIP treatment. In agreement, H3K9me3 increased in defined foci following treatment (Fig. 6A). We further characterised and quantified this observation with high-content screening. Fluorescent intensity was used as a readout for the level of each marker in cells grown under normal conditions and upon treatment with TIP (Supplementary Fig. 16A). Treatment with TIP led to a decrease in active transcription markers by 35% and 10% for H3K9ac and H3K27ac, respectively, and an increase in the repressive marker H3K9me3 by 100% (Supplementary Fig. 16B).

To determine if these changes to the chromatin correspond to changes in accessibility, nuclease assays were performed in the presence and absence of TIP (Fig. 6B). Nuclease activity was monitored by qPCR at three genes, *VASP* and *PTEN* which are lose RNAPII binding following TIP treatment, and *HBB* which represents an inactive gene in a closed chromatin region. Under normal conditions, *VASP* and *PTEN* are digested and therefore represent accessible genes. TIP halts the digestion and therefore alters the chromatin state to a closed form. This is consistent with a loss of RNAPII and a change in chromatin marks. As expected, *HBB* was essentially not digested and TIP had a negligible impact.

To assess the overall impact on cell function, we performed live-cell growth assays under normal and MVI transient knockdown conditions. A threefold decrease in growth was observed following knockdown (Fig. 6C, D and Supplementary Fig. 17A). The increase in growth rate after 3 days is consistent with the end of the transient knockdown. The cell number over the previous 3 days was essentially constant with no visual increase in cell death compared to the transfected mock. Moreover, the cells do not show any visible signs of stress (Supplementary Fig. 8B). Overall, this change is indicative of a larger cellular response to the perturbation of RNAPII and the resulting decrease in gene expression.

**Fig. 7:**
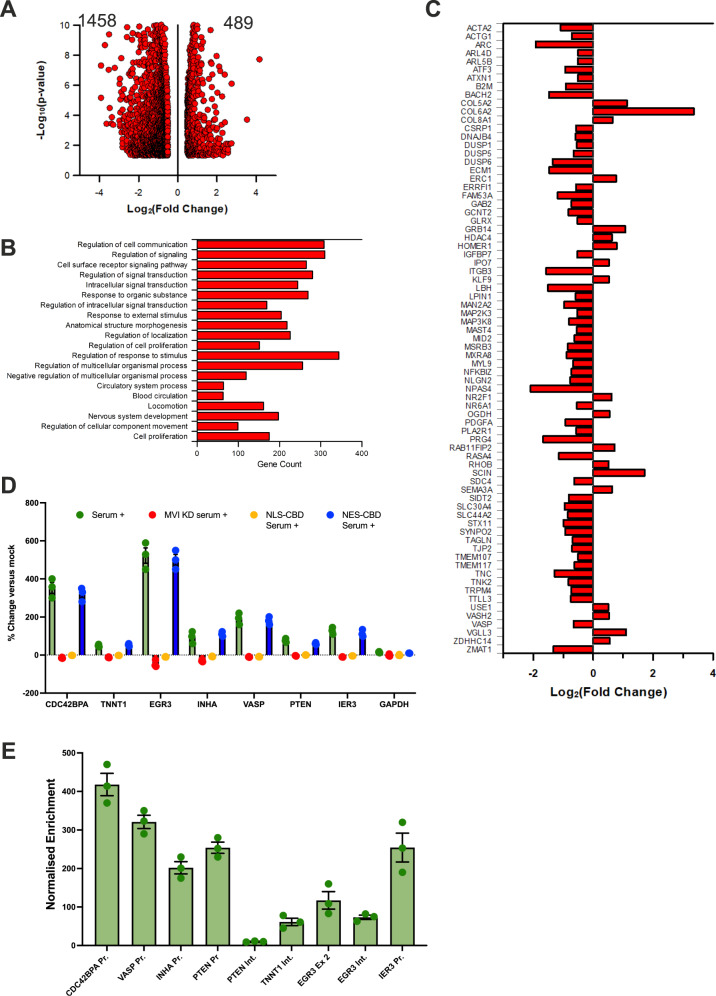
Myosin VI dependent changes in gene expression. **A** Volcano plot of differentially-expressed genes from RNA-seq following MVI knockdown. *p* values are calculated using the Wald test. Western blots are shown in Supplementary Fig. 17 for the MVI knockdown. **B** GO terms, with gene count, for Biological Process corresponding to the genes negatively expressed following MVI knockdown. GO terms are plotted based on significant enrichment, as shown in Supplementary Table 1. **C** Plot of gene expression changes for serum-responsive genes within the list of differentially-expressed genes following MVI knockdown. **D** RT-qPCR Gene expression analysis of five serum-responsive genes and housekeeping gene *GAPDH* with treatment of serum alone, serum following MVI KD, and serum following transfection with either NLS-CBD or NES-CBD (See Supplementary Fig. 17 for western-blot for knockdown). Data are plotted relative to non-stimulated expression from three independent experiments. Error bars represent SEM from three independent experiments. **E** MVI ChIP against labelled loci. Values are the average of three independent experiments. Error bars represent SEM from three independent experiments.

To explore the global changes in gene expression which arise from the perturbations above, RNA-seq measurements were performed under normal and MVI-knockdown conditions. In total, we observed a significant change in the expression of 1947 genes following knockdown. From this set, 489 genes were upregulated and 1458 were down-regulated (Fig. 7A), which highlights the negative impact on transcription due to the loss of MVI (Supplementary Fig. 17B). This is consistent with the STORM data that demonstrated disruption of the spatial organisation of RNAPII under these conditions (Fig. 3A, B). The down-regulated genes were taken forward to Gene Ontology (GO) analysis. The breakdown for GO Biological Process reveals that the majority of the processes affected are coupled to cell response pathways, rather than to housekeeping ones (Fig. 7B and Supplementary Table 1). Hence, disruption of MVI perturbs expression of specific genes, but it does not completely halt transcription.

We observed that transcription stimulation with serum had a significant impact on the nuclear organisation of both MVI and RNAPII (Figs. 1, 2 and Supplementary Fig. 5). Interestingly, the RNA-seq data also revealed that 75 of the differentially-expressed genes are known to be serum responsive^38^. Of these genes, 56 were significantly down-regulated (Fig. 7C). To investigate this impact further, we performed serum stimulation on cells where MVI had been knocked down (Supplementary Fig. 17). We then used RT-qPCR to monitor the expression of 5 example serum-responsive genes and housekeeping gene *GAPDH*. Control measurements showed that serum stimulation increases the expression of the target genes (Fig. 7D). In all cases, knockdown of MVI completely abrogated this response. To confirm the nuclear-specific role of MVI in this process, we utilised the NLS-CBD construct which acts as a dominant-negative competitor in the nucleus and leads to perturbed RNAPII behaviour (Supplementary Fig. 12, Fig. 5). As a control, we used the NES-CBD construct which only perturbs cytoplasmic function^29–32^. Transfection with the NLS-CBD yielded the same response as siRNA-treatment, NES-CBD was equivalent to wild type (Fig. 7D). Overall, nuclear perturbation of myosin VI impacted the serum-response. Lastly, ChIP of MVI confirmed its binding at the promoters corresponding to these genes (Fig. 7E).

Taken together, the data show that perturbation of MVI, which impacts the spatial organisation of RNAPII, impedes gene expression under stimulatory conditions. Therefore, MVI is critical for the cell’s response to stimulus.

### Myosin VI acts as a molecular anchor

**Fig. 8:**
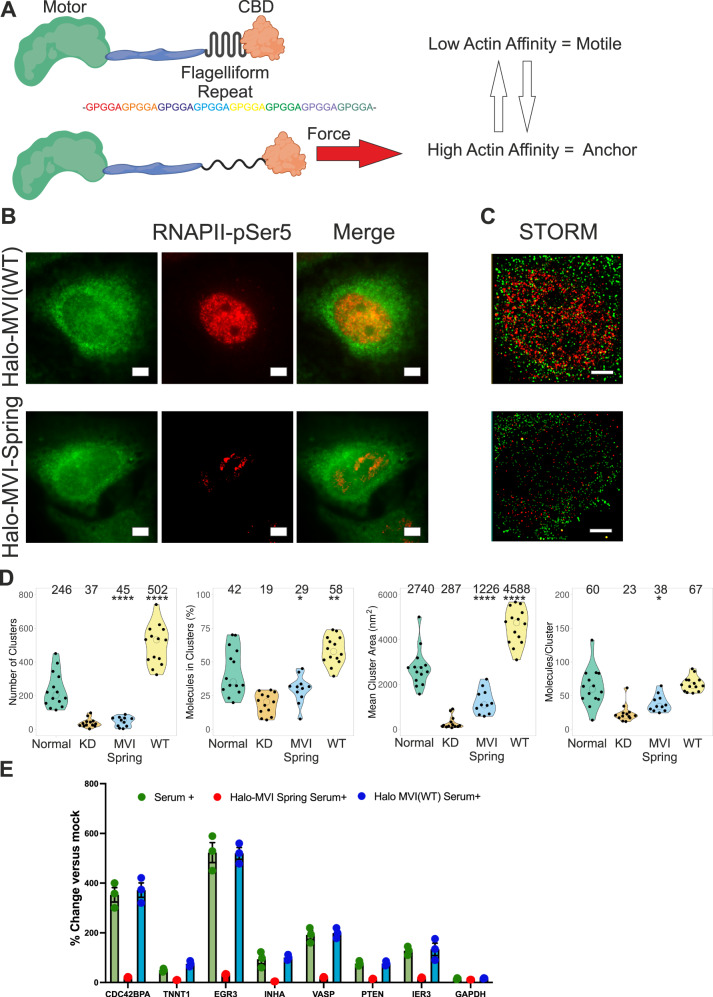
Myosin VI anchors RNAPII at transcription sites. **A** Cartoon depiction of MVI containing the molecular spring (Flagelliform repeat) inserted proximal to the CBD. At low force, the spring is folded and MVI is in a low actin affinity mode. The application of force leads to extension of the spring which triggers the high affinity actin binding mode. **B** Example widefield imaging of Halo-MVI (Wild Type) and Halo-MVI-spring stained with JF549 (green) and corresponding Immunofluorescence staining against RNAPII-pSer5 (red) in HeLa cells (Scale bar 5 μm) following MVI knockdown (see Supplementary Fig. 18E for western-blot control for knockdown). Further example images are in Supplementary Figs. 19, 20. **C** Example STORM render image of MVI (green) and RNAPII-pSer5 (red) following transfection of Halo-MVI and Halo-MVI-spring, as described in the methods (scale bar 2 μm). **D** Cluster analysis of RNAPII-pSer5 nuclear organisation following treatment in (**C**). WT refers to Halo-MVI transfection. Individual data points correspond to the average value for a cell ROI. (*n* = 14 for Normal, 13 for KD, 11 for MVI Spring and 14 for WT) The values represent the mean from the ROIs for each condition (Only statistically significant changes are highlighted *p < 0.05; **p < 0.01; ****p < 0.0001 by two-tailed *t* test compared to normal conditions). **E** RT-qPCR Gene expression analysis of 5 serum-responsive genes and housekeeping gene *GAPDH* with treatment of serum alone, serum following MVI knockdown (MVI KD and transfection with either Halo MVI (WT) or Halo MVI Spring. Data are plotted relative to non-stimulated expression from three independent experiments. Error bars represent SEM from three independent experiments.

We have revealed that MVI is a key regulator of RNAPII spatial organisation. However, the mechanism governing how MVI achieves this fundamental role is unknown. We hypothesised that MVI is bound to chromatin and/or transcription regulators through its CBD, and to RNAPII through actin^6^. MVI is a rare motor protein with the ability to switch from a motile state to a molecular anchor, when forces greater than 2pN are applied to the molecule^20^. RNAPII is a large macromolecular machine which could diffuse or potentially move along DNA, away from transcription initiation sites. Such a movement would apply load upon MVI and then trigger the motor protein to anchor RNAPII in situ. To test this hypothesis, we set out to disrupt the ability of MVI to respond to force. To achieve this, we inserted a molecular spring consisting of a repeated penta-peptide sequence from spider-silk flagelliform into the MVI tail (Fig. 8A). The repeat sequence has been widely used as a calibrated tension sensor^39^. The spring unfolds as tension up to 10pN is exerted across the molecule^39^, thereby preventing load-induced changes on MVI. Therefore, this MVI construct should not be responsive to force up to 10pN.

We first explored the impact of the insertion upon the biochemical properties of MVI. Firstly, CD spectroscopy confirmed that the recombinant protein is folded and stable (Supplementary Fig. 18A), similar to wild type (WT) MVI (Supplementary Fig. 18B). Moreover, the actin-activated ATPase activity was not affected by the presence of the insert, with *k*_cat_ 5.9 s^−1^ and 5.5 s^−1^ for MVI spring and WT, respectively (Supplementary Fig. 18C). To assess whether the spring insert disrupts the load-induced anchoring ability in MVI, we then compared the ATPase rate of two stable dimeric constructs of the protein, one containing and one lacking the insert. The stable dimeric MVI, in which a leucine zipper replaces the MVI C-terminal domain at residue 920 to dimerise the protein, has been widely used in MVI studies^9,40^. We first confirmed that the spring dimeric construct is indeed dimeric, as shown by a similar size-exclusion chromatography elution profile to WT dimeric MVI (Supplementary Fig. 18D).

As previously observed^41^, the dimeric form of MVI displayed gating ATPase activity, whereby only one motor domain within the dimer hydrolyses ATP at a given point. Therefore, the observed ATPase rate was half of that of the monomeric MVI (*k*_cat_ 2.84 s^−1^), as seen in Supplementary Fig. 18C. This gating behaviour results from load-induced conformation changes: the conformation of the leading motor is in a state which remains bound to actin^20^. However, the insertion of the spring within the dimer construct showed an ATPase rate 4.9 s^−1^, which is similar to that of the monomeric MVI (Supplementary Fig. 18C). This suggests the two motor domains are functioning independently. We propose that the spring-induced flexibility and the resulting inability to respond to load prevents the communication between the leading and the rear motor domains within the dimer.

Having assessed the biochemical properties of the spring construct, we transiently over-expressed it into mammalian cells carrying a Halo-Tag where endogenous MVI had been knocked down. As a control, we over-expressed Halo-WT-MVI (Fig. 8B and Supplementary Fig. 18E). Both constructs were localised throughout the cytoplasm and the nucleus (Fig. 8B, Supplementary Figs. 19, 20). Using STORM (Fig. 8C), it was possible to observe clusters of both proteins in the nucleus (Supplementary Fig. 21). Over-expressed WT MVI had an enhanced clustering behaviour compared to endogenous protein. While, the spring construct displayed a similar number of clusters but the area and number of molecules per cluster were significantly smaller. This implies that the clustering behaviour of MVI has a force-dependence.

We then assessed the ability of the spring construct to rescue the disrupted RNAPII nuclear organisation. Unlike WT MVI (Fig. 8B–D, the spring construct was unable to fully rescue the RNAPII nuclear distribution and clustering. These results suggest that the ability of MVI to respond to force is required for rescuing the disrupted RNAPII distribution. In further support of this, the over-expression of WT MVI was not only able to rescue RNAPII distribution, but to also increase RNAPII clusters number, size and percentage of molecules in clusters, along with co-localisation (Fig. 8C, D and Supplementary Fig. 20B). Lastly, we revisited the RT-qPCR analysis on the serum-responsive genes where we knocked-down endogenous MVI followed by transfection with WT MVI or spring (Fig. 8E). The inability of the spring construct to rescue RNAPII localisation corresponds to a significant loss in gene expression. Therefore, we propose that the force-induced anchoring ability of MVI is critical for the nuclear organisation of RNAPII and that MVI physically holds RNAPII at sites of transcription initiation.

## Discussion

Following a multidisciplinary approach, we have been able to shed light on to the regulation of transcription by addressing how myosins contribute to the spatial organisation of transcription. We have observed that the molecular motor MVI is clustered within the nucleus and we showed that this activity is linked to the spatial organisation of RNAPII into transcription clusters. We have also been able to show that the spatial and dynamic changes in RNAPII behaviour are dependent upon MVI and relate to wider chromatin and transcriptome changes.

For over a decade, MVI has been linked to transcription^6–9,22^ and here we have gained further understanding of its nuclear function, including its interaction with nuclear receptors and DNA^6^. Furthermore, until now, it has not been possible to determine the precise role that MVI plays in this vital process and why the properties of a myosin would be required for transcription. Here, we have dissected the molecular mechanistic need for MVI to be capable of acting as a molecular anchor in a force-induced manner. Therefore, we have been able to directly address why a nuclear myosin, with its biophysical properties to sense and respond to force, is required in transcription in order to hold RNAPII in situ. Overall, it would not be surprising if nuclear myosins are deployed in a similar way in other nuclear processes such as DNA repair, where myosin proteins are also known to function^42–45^.

The study of nuclear myosins is challenging due to the variety of cytoplasmic functions. However, we have implemented an array of approaches to perturb myosin with in the nucleus. This has included using siRNA, small-molecule inhibition and the use of nuclear targeted dominant-negative constructs. Moreover, targeting of nuclear MVI and nuclear actin leads to a similar impact upon RNAPII. We also found that small-molecule inhibition perturbs nuclear MVI organisation and dynamics, which correlates with changes in RNAPII organisation. Likewise, disruption of RNAPII led to changes in nuclear MVI dynamics which suggests that there is an interconnected relationship between the two proteins. Our dominant-negative experiments also used cytoplasmic-restricted versions which did not lead to the changes in RNAPII organisation or gene expression. Moreover, we found that the nuclear fraction of MVI increased upon transcription stimulation and decrease during TIP treatment. This leads us to conclude that perturbation of cytoplasmic MVI would not bring about these changes. Lastly, MVI knockdown or inhibition did not cause obvious defects in the cytoskeleton which could also impact the nuclear organisation. This was also consistent with our single-molecule tracking experiments using an isolated SNAP-tag reporter. Here, we found that diffusion of the reporter was identical even upon MVI perturbation therefore we conclude that the nuclear environment is not altered non-specifically.

**Fig. 9:**
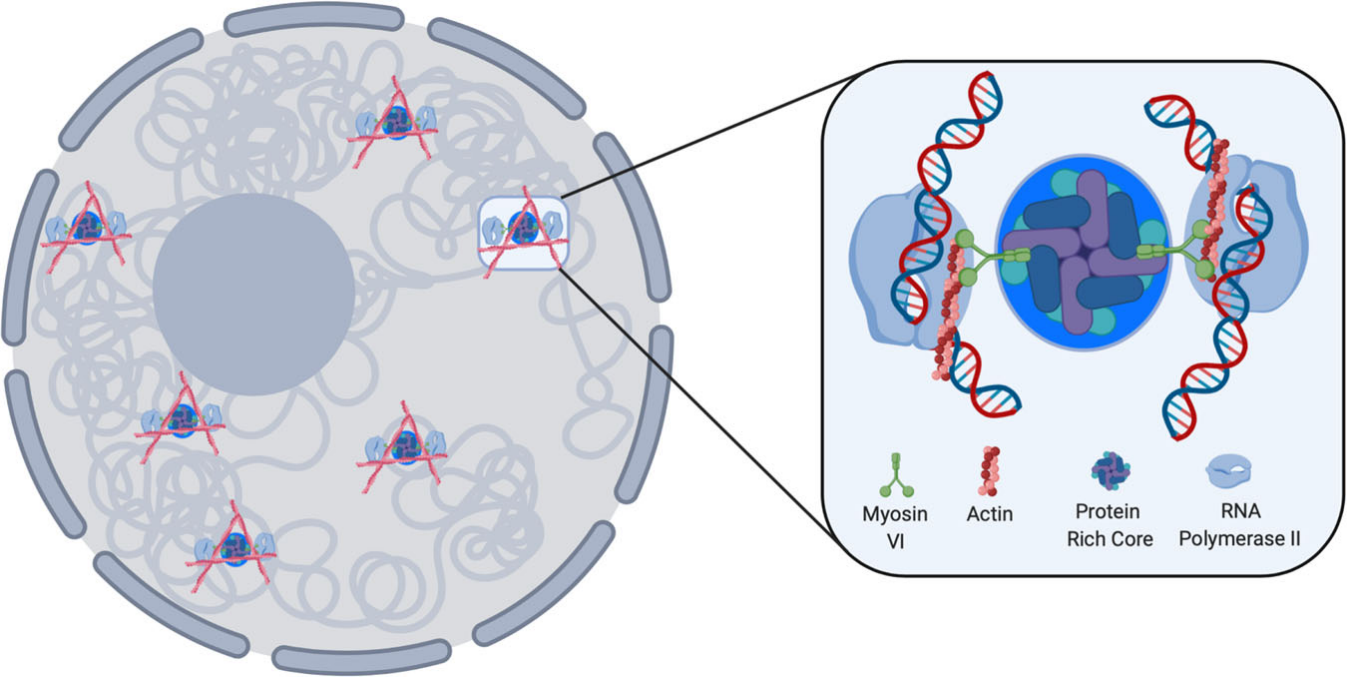
Schematic of myosin VI anchoring RNAPII at transcription factories. Based on the data presented here we can propose this model for RNAPII clustering. Transcription sites within the nucleus are marked by clustering of RNAPII and actin filaments. Focusing on the clusters: The protein rich core consists of transcription regulators which the DNA would also contact. MVI interacts with these proteins and DNA via its C-terminal domain. MVI then interacts with RNAPII through nuclear actin filaments thereby providing the framework for mechanical linkage to RNAPII.

Overall, we present a model, whereby MVI anchors RNAPII at, or near, sites of transcription initiation (Fig. 9) within the nucleus. This approach could enable enhanced RNAPII binding to initiate transcription and facilitate rapid recycling of RNAPII to drive higher expression levels upon transcription stimulation. Such a mechanism would also fit with the observation that MVI functions in gene pairing^46^. We have previously shown that MVI interacts with RNAPII through actin^6^, present within the RNAPII complex. Given that the interaction of MVI with nuclear receptors and DNA is mediated by its CBD, we speculate that MVI could be bound to chromatin and transcription regulators via the CBD at the factory core, whilst simultaneously interacting with RNAPII at the motor domain through actin. The exact structure of the complex and the network of interactions needs to be defined. We propose that the MVI orientation is critical to enable force-induced anchoring of RNAPII. These results are consisted with recent reports of nuclear actin clustering and filaments forming during transcription stimulation^34^. Interestingly, MVI is the only actin minus-end motor protein, therefore actin polymerising from the sites of transcription would also provide a framework to recruit MVI to these sites and subsequently to RNAPII.

Upon perturbation of MVI, RNAPII was observed around the nuclear periphery, which highlights the key role of MVI in maintaining the nuclear organisation of RNAPII. The basis for this localisation is not yet clear, but we can postulate two mechanisms. The protein may be en-route for nuclear export as part of a degradation process which initiates once transcription is disrupted^47^. Alternatively, RNAPII may be excluded from the chromatin body as DNA condensation takes place, through the increase of repressive transcription histone marks. Moreover, an increase in inaccessible heterochromatin would further increase RNAPII dynamics, by reducing RNAPII interactions with DNA.

The ability of RNAPII to cluster has been shown to be important for transcription^10,19^. Our findings further support this, given the disruptive effect of MVI perturbation on the organisation of RNAPII and, subsequently, on transcription. Clustering of RNAPII has been shown to be dynamic with structures lasting around 10 s^10,19^ and MVI has also been shown to display dynamic binding (up to 20 s)^9^. We therefore conclude that there is likely to be turnover of proteins within these clusters, however we suggest that MVI enhances the RNAPII binding time to enable transcription initiation. Phase separation drives the formation of membraneless compartments through cooperative interactions between molecules^48^. This process has been shown to contribute to the regulation of transcription, with impacts upon enhancers, mediators and the RNAPII C-Terminal-Domain^13,49,50^. In contrast, our results suggest there are underlying mechanical processes contributing to the establishment of transcription clusters with regard to RNAPII. Importantly, phase separation observations are still consistent with our conclusions because these processes can occur locally within the clusters and may contribute to larger genomic rearrangements by bringing chromatin to the factories.

According to our model, we expect the need for myosins to be deployed in conditions of high transcriptional load to increase activate and/or recycling of RNAPII. Indeed, our RNA-seq analysis suggests that MVI plays a key role during stimulation and the downstream cell response processes. We therefore propose that the function of MVI is particularly critical when cells are under high transcription load and undergo rapid changes in the gene expression landscape. This is consistent with previous observations of MVI being over-expressed in several cancers, along with interacting with nuclear receptors and participating in the expression of target genes^6,51–53^ to drive adaptation.

## Methods

### Constructs

A list of constructs are provided in Supplementary Table 2. Constructs generated in this work are described below. Halo or SNAP tags were used through to provide a specific protein labelling strategy for live cells^54^. The SNAP-Rpb1 construct was generated by sub-cloning the SNAP tag from the pSNAP_f_-C1 plasmid (Addgene 58186) into the NheI and SacII of the pHalo-Rbp1 plasmid (A gift from Darzacq lab), following removal of the Halo tag. pcDNA3.1 Halo-MVI, pcDNA3.1 Halo-MVI-Spring, pFastbac Halo-MVI-Spring, and pFastbac Halo-MVI-Spring bZip, pEGFP-NES-Motor and pEGFP-NES-CBD were ordered as synthetic constructs.

### Protein expression using baculovirus system

Full-length MVI NI, MVI Spring, MVI Spring bZip (1-914) and *Xenopus* calmodulin were expressed in *Sf9* and *Sf21* (*Spodoptera frugiperda*) insect cells using the Bac-to-Bac^®^ Baculovirus Expression System (Invitrogen). *Sf9* cells were cultured in Sf900 media (Gibco). Recombinant bacmids were generated following the manufacturer’s instructions and were transfected into adherent Sf9 cells to generate the P1 viral stock. *Sf9* cells were infected in suspension at 27 °C and 100 rpm with 1 in 50 dilution of P1 and P2 viral stocks to yield P2 and P3 stocks, respectively. Finally, expression of recombinant proteins was set up by infecting *sf21* cells with the P3 viral stock in Spodopan media (PAN Biotech). To ensure correct folding of the myosin VI constructs, cells were simultaneously infected with P3 viral stock of the myosin VI constructs together with calmodulin at a 0.75 ratio The cells were harvested after 3 days by centrifugation for 15 min at 700 × *g* and at 4 °C and resuspended in ice cold myosin extraction buffer (90 mM KH_2_PO_4_, 60 mM K_2_HPO_4_, 300 mM KCl, pH 6.8), supplemented with Proteoloc protease inhibitor cocktail (Expedeon) and 100 µM PMSF, before proceeding to protein purification. Prior to sonication, an additional 5 mg recombinant calmodulin was added together with 2 mM DTT. After sonication, 5 mM ATP and 10 mM MgCl_2_ were added and the solution was rotated at 4 °C for 30 min before centrifugation (20,000 × *g*, 4 °C, 30 min). Then, the cell lysate was subjected to the purification. Proteins were purified by affinity chromatography (HisTrap FF, GE Healthcare). The purest fractions were further purified through a Superdex 200 16/600 column (GE Healthcare).

### Cell culture and transfection

HeLa (ECACC 93021013) cells were cultured at 37 °C and 5% CO_2_, in Gibco MEM Alpha medium with GlutaMAX (no nucleosides), supplemented with 10% Fetal Bovine Serum (Gibco), 100 units/ml penicillin and 100 µg/ml streptomycin (Gibco). For the transient expression of myosin VI and mutants, HeLa cells grown on glass coverslips were transfected using Lipofectamine 2000 (Invitrogen), following the manufacturer’s instructions. Depending on the construct, 24–72 h after transfection, cells were subjected to nuclear staining using Hoechst 33342 (Thermo Scientific), fixed and analysed or subjected to indirect immunofluorescence (see below).

### Cell treatments

For MVI knockdown experiments, HeLa cell monolayers, seeded to 30–50% confluency, were transfected with human myosin VI siRNA duplex (5′GGUUUAGGUGUUAAUGAAGtt-3′) (Ambion) or AllStars Negative Control siRNA duplex (Qiagen) at a concentration of 50 nM, using Lipofectamine 2000 (Invitrogen), according to the manufacturer’s guidelines. Cells were fixed or harvested after 48 h for further analysis. To inhibit myosin VI, cells were treated with 25 µM TIP (Sigma) for 1 h at 37 °C. To inhibit actin polymerisation, cells were treated with 1 µM Latrunculin B (Sigma) for 1 h at 37 °C. To inhibit RNAPII transcription, cells were treated with 5 µg/ml α-amanitin (Sigma) for 4 h at 37 °C. For serum stimulation, 4.8 × 10^5^ Hela cells were seeded in DMEM complete media in 6-well plates to achieve 70–80% confluency on the following day. For serum starvation, cells were grown in DMEM with 0.5% FBS at 37 °C for 24 h. To stimulate the starved cells, media was replaced with complete media containing 10% FBS for another 24 h after which cells were fixed for immunofluorescence.

### Stable cell line generation

The stable cell lines used in this study are named as HeLa-Halo MVI (pHalo-MVI vector stably expressed in HeLa) and Hela-Halo Rpb1 (pHalo-Rpb1 vector stably expressed in HeLa). The Hela-Halo MVI were generated as described in^9^. To generate Hela cells stably expressing pHalo-Rpb1, the plasmid was transfected in 6-well plates using lipofectamine 2000 protocol (Thermo Fisher Scientific). The transfected cells were selected using optimal concentrations of G418 antibiotic (G418 Sulfate, Gibco) in the complete media (0.5 mg/ml) for 9–10 days until most of the untransfected cells were dead and those survived would have integrated the desired plasmid. The cells were harvested when they reached about 60–70% confluency and were expanded into multiple T75 flasks with 1:10 ratio. Some cells at this stage were seeded onto coverslips and stable transfection of desired plasmids was confirmed by using specific fluorescent ligands to Halo-tag (TMR, Promega). The cells seeded in T75 flasks were allowed to grow for further 3–4 weeks in complete media with G418 replaced twice a week. When the cells reached high confluency, they were frozen down as polyclonal stable cell line.

### Immunofluorescence

Transfected and non-transfected HeLa cells were fixed for 15 min at room temperature in 4% (w/v) paraformaldehyde (PFA) in PBS and residual PFA was quenched for 15 min with 50 mM ammonium chloride in PBS. All subsequent steps were performed at room temperature. Cells were permeabilised and simultaneously blocked for 15 min with 0.1% (v/v) Triton X-100 and 2% (w/v) BSA in PBS. Cells were then immuno-stained against the endogenous proteins by 1 h incubation with the indicated primary and subsequently the appropriate fluorophore-conjugated secondary antibody (details below), both diluted in 2% (w/v) BSA in PBS. When using anti-phospho antibodies, immunofluorescence protocol was performed in TBS. The following antibodies were used at the indicated dilutions: Rabbit anti-myosin VI (1:200, Atlas-Sigma HPA0354863), Rabbit anti-Histone H3 (tri methyl K9) (1:500, Abcam ab8898), Rabbit anti-Histone H3 (acetyl K27) (1:500, Abcam ab4729), Rabbit anti-Histone H3 (acetyl K9) (1:200, Abcam ab4441), Rabbit anti-RNAPII phospho Ser5 (1:500, Abcam Ab5131), Mouse anti-RNAPII phospho Ser5 (1:500, Abcam Ab5408), Rabbit anti-LB1 (1:200, Abcam ab16048) Donkey anti-mouse Alexa-Fluor 488-conjugated (1:500, Abcam Ab181289), Donkey anti-rabbit Alexa-Fluor 647-conjugated (1:500, Abcam Ab181347) and Donkey anti-rabbit Alexa-Fluor 488-conjugated antibody (1:500, Abcam Ab181346). Coverslips were mounted on microscope slides with Mowiol (10% (w/v) Mowiol 4–88, 25% (w/v) glycerol, 0.2 M Tris-HCl, pH 8.5), supplemented with 2.5% (w/v) of the anti-fading reagent DABCO (Sigma).

### Immunoblot analysis

The total protein concentration was determined by Bradford Assay (Sigma) following the manufacturer’s instructions. Cell lysates were heat-denatured and resolved by SDS-PAGE. The membrane was probed against the endogenous proteins by incubation with primary Rabbit anti-myosin VI (1:500, Atlas-Sigma HPA0354863-100UL) or Mouse anti-RNAPII phospho Ser5 (1:500, Abcam Ab5408) and subsequently secondary Goat anti-rabbit antibody (1:15000 Abcam ab6721) or Goat anti-mouse antibody (1:15000, Abcam ab97023) coupled to horseradish peroxidase. The bands were visualised using the ECL Western Blotting Detection Reagents (Invitrogen) and the images were taken using Syngene GBox system. Images were processed in ImageJ.

### Fluorescence imaging

Cells were visualised using either the ZEISS LSM 880 confocal microscope or the widefield Olympus IX71 microscope. The former was equipped with a Plan-Apochromat 63 × 1.4 NA oil immersion lens (Carl Zeiss, 420782-9900-000). Three laser lines, i.e. 405, 488 and 561 nm, were used to excite the fluorophores, i.e. Hoechst, GFP and RFP, respectively. The built-in dichroic mirrors (Carl Zeiss, MBS-405, MBS-488 and MBS-561) were used to reflect the excitation laser beams on to cell samples. The emission spectral bands for fluorescence collection were 410–524 nm (Hoechst), 493–578 nm (GFP) and 564–697 nm (RFP). The detectors consisted of two multi anode photomultiplier tubes (MA-PMT) and 1 gallium arsenide phosphide (GaAsP) detector. The green channel (GFP) was imaged using GaAsP detector, while the blue (Hoechst) and red (RFP) channels were imaged using MA-PMTs. ZEN software (Carl Zeiss, ZEN 2.3) was used to acquire and render the confocal images. The later was equipped with an PlanApo 100xOTIRFM-SP 1.49 NA lens mounted on a PIFOC z-axis focus drive (Physik Instrumente, Karlsruhe, Germany), and illuminated with an automated 300 W Xenon light source (Sutter, Novato, CA) with appropriate filters (Chroma, Bellows Falls, VT). Images were acquired using a QuantEM (Photometrics) EMCCD camera, controlled by the Metamorph software (Molecular Devices). The whole volume of cells was imaged by acquiring images at z-steps of 200 nm. Widefield images were deconvolved with the Huygens Essential version 17.10 software. Confocal Images were deconvolved using the Zeiss Zen2.3 Blue software, using the regularised inverse filter method. All images were then analysed by ImageJ.

### STORM imaging

Cells were seeded on pre-cleaned No 1.5, 25 mm round glass coverslips, placed in 6-well cell culture dishes. Glass coverslips were cleaned by incubating them for 3 h, in etch solution, made of 5:1:1 ratio of H_2_O: H_2_O_2_ (50 wt. % in H_2_O, stabilised, Fisher Scientific): NH_4_OH (ACS reagent, 28–30% NH_3_ basis, Sigma), placed in a 70˚C water bath. Cleaned coverslips were repeatedly washed in filtered water and then ethanol, dried and used for cell seeding. Transfected or non-transfected cells were fixed in pre-warmed 4% (w/v) PFA in PBS and residual PFA was quenched for 15 min with 50 mM ammonium chloride in PBS. Immunofluorescence (IF) was performed in filtered sterilised PBS, unless when anti-phospho antibodies were used. Then, IF was performed in filtered sterilised TBS. Cells were permeabilized and simultaneously blocked for 30 min with 3% (w/v) BSA in PBS or TBS, supplemented with 0.1% (v/v) Triton X-100. Permeabilized cells were incubated for 1 h with the primary antibody and subsequently the appropriate fluorophore-conjugated secondary antibody, at the desired dilution in 3% (w/v) BSA, 0.1% (v/v) Triton X-100 in PBS or TBS. The antibody dilutions used were the same as for the normal IF protocol (see above), except from the secondary antibodies which were used at 1:250 dilution. Following incubation with both primary and secondary antibodies, cells were washed 3 times, for 10 min per wash, with 0.2% (w/v) BSA, 0.05% (v/v) Triton X-100 in PBS or TBS. Cells were further washed in PBS and fixed for a second time with pre-warmed 4% (w/v) PFA in PBS for 10 min. Cells were washed in PBS and stored at 4 °C, in the dark, in 0.02% NaN3 in PBS, before proceeding to STORM imaging.

Before imaging, coverslips were assembled into the Attofluor^®^ cell chambers (Invitrogen). Imaging was performed in freshly made STORM buffer consisting of 10% (w/v) glucose, 10 mM NaCl, 50 mM Tris—pH 8.0, supplemented with 0.1% (v/v) 2-mercaptoethanol and 0.1% (v/v) pre-made GLOX solution which was stored at 4 °C for up to a week (5.6% (w/v) glucose oxidase and 3.4 mg/ml catalase in 50 mM NaCl, 10 mM Tris—pH 8.0). All chemicals were purchased from Sigma.

Imaging was undertaken using the Zeiss Elyra PS.1 system. Illumination was from a HR Diode 642 nm (150 mW) and HR Diode 488 nm (100 mW) lasers where power density on the sample was 7–14 and 7–12 kW/cm^2^, respectively

Imaging was performed under highly inclined and laminated optical (HILO) illumination to reduce the background fluorescence with a 100× NA 1.46 oil immersion objective lens (Zeiss alpha Plan-Apochromat) with a BP 420-480/BP495-550/LP 650 filter. The final image was projected on an Andor iXon EMCCD camera with 25 ms exposure for 20000 frames.

Image processing was performed using the Zeiss Zen Black software. Where required, two channel images were aligned following a calibration using a calibration using pre-mounted MultiSpec bead sample (Carl Zeiss, 2076-515). For calibration, a 2 μm Z stack was acquired at 100 nm steps. The channel alignment was then performed in the Zeiss Zen Black software using the Affine method to account for lateral, tilting and stretching between the channels. The calibration was performed during each day of measurements.

The images were then processed through our STORM analysis pipeline using the Zeiss Zen Black software. Single-molecule detection and localisation was performed using a 9 pixel mask with a signal to noise ratio of 6 in the “Peak finder” settings while applying the “Account for overlap” function. This function allows multi-object fitting to localise molecules within a dense environment. Molecules were then localised by fitting to a 2D Gaussian.

The render was then subjected to model-based cross-correlation drift correction and detection grouping to remove detections within multiple frames. Typical localisation precision was 20 nm for Alexa-Fluor 647 and 30 nm for Alexa-Fluor 488. The final render was then generated at 10 nm/pixel and displayed in Gauss mode where each localisation is presented as a 2D gaussian with a standard deviation based on its precision. The localisation table was exported as a csv for import in to Clus-DoC.

### Clus-DoC

The single-molecule positions were exported from Zeiss Zen Black version and imported into the Clus-DoC analysis software^21^ (https://github.com/PRNicovich/ClusDoC). The region of interest was determined by the nuclear staining. First the Ripley K function was completed on each channel identifying the r max. The r max was then assigned for DBSCAN if one channel was being analysed or Clus-Doc if two channel colcalisation was being analysed. The MinPts was 3 and a cluster required 10 locations, with smoothing set at 7 nm and epsilon set at the mean localisation precision for the dye. All other analyses parameters and colocalization thresholds remained at default settings^21^. Data concerning each cluster was exported and graphed using Plots of Data.

### High-content imaging

Cells were seeded onto Corning^®^ 384 well microplates at a density of 5000 cells per well. The cells were grown for 24 h, followed by the necessary treatments. The cells were fixed and immunofluorescence was undertaken as described above, due to the cells being grown directly on the plates no mounting of coverslips was required. Stained cells in plate were scanned via Cellomics ArrayScan™ XTI High-Content Analysis (HCS) platform (Thermo Fisher Scientific), with a 20× Objective. Compartment Analysis Bio Application software (Cellomics) was applied to quantitatively analyse the immunostaining spots in the nucleus based on a mask created using the nuclear Hoechst staining. For each experiment, at least 1000 valid single cells per culture well were quantified and at least ten independent culture wells (ten biological replicates) were analysed, fluorescence intensities were then plotted using Prism 8, Graphpad.

### Size-exclusion chromatography

100 μl samples of 2 mg/ml purified protein, was applied to a Superdex 200 (30 × 1 cm) analytical column (GE Healthcare) equilibrated in 150 mM NaCl, 50 mM Tris.HCl (pH 7.5) and 1 mM DTT and controlled using Waters 626 HPLC and OMNISEC (Malvern Panalytical) at room temperature.

### Multifocal imaging and particle tracking analysis

Cells stably or transiently expressing Halo-tag or SNAP-tag constructs were labelled for 15 min with HaloTag-JF549 or SNAP-tag-JF549 ligand, respectively, in cell culture medium at 37 °C, 5% CO_2_. 10 nM ligand was used to label Halo-tagged myosin VI constructs, whereas 50 nM ligand was used to label Halo- or SNAP-tagged RNAPII. Cells were washed for 3 times with warm cell culture medium and then incubated for further 30 min at 37 °C, 5% CO_2_. Cells were then washed three times in pre-warmed FluoroBrite DMEM imaging medium (Thermo Fisher Scientific), before proceeding to imaging.

Single-molecule imaging was performed using an aberration-corrected multifocal microscope (acMFM), as described by Abrahamsson et al.^36^. Samples were imaged using 561 nm laser excitation, with typical irradiance of 4–6 kW/cm^2^ at the back aperture of a Nikon 100 × 1.4 NA objective. Images were relayed through a custom optical system appended to the detection path of a Nikon Ti microscope with focus stabilisation. The acMFM detection path includes a diffractive multifocal grating in a conjugate pupil plane, a chromatic correction grating to reverse the effects of spectral dispersion, and a nine-faceted prism, followed by a final imaging lens.

The acMFM produces nine simultaneous, separated images, each representing successive focal planes in the sample, with ca. 20 µm field of view and nominal axial separation of ca. 400 nm between them. The nine-image array is digitised via an electron multiplying charge coupled device (EMCCD) camera (iXon Du897, Andor) at up to 32 ms temporal resolution, with typical durations of 30 s. Microscope control and data acquisition was through Nikon NIS-Elements.

3D + t images of single molecules were reconstructed via a calibration procedure, implemented in Matlab (MathWorks), that calculates and accounts for: (1) the inter-plane spacing, (2) affine transformation to correctly align each focal plane in the xy plane with respect to each other, and (3) slight variations in detection efficiency in each plane, typically <±5–15% from the mean.

Reconstructed data were then subject to pre-processing, including background subtraction, mild deconvolution (3–5 Richardson-Lucy iterations), and/or Gaussian de-noising prior to 3D particle tracking using the MOSAIC software suite^55^. Parameters were set where maximum particle displacement was 400 nm and a minimum of 10 frames was required. Tracks were reconstructed, and diffusion constants were extracted via MSD analysis^56^ using custom Matlab software assuming an anomalous diffusion model. Code is available upon request to the corresponding author.

### Circular dichroism spectroscopy

1 mg mL^−1^ of protein was analysed using far UV spectra (190–270 nm) measured by a Jasco J715 Circular Dichroism Spectrometer (Jasco Inc.). Spectra were taken at 20 °C. 4 readings were taken for each measurement and averaged by the software provided.

For spectra analysis the following Eq. 1 was used.

1 $${\left[\theta \right]}_{{MRW}\,=\,}\frac{\frac{{MW}}{(n\,-\,1)}\,\times \,\theta }{l\,\times\, c\,\times\, 10}$$

where *θ*_*MRW*_ is the mean residue elipticity, *MW* is the molecular weight of the protein, *n* is the number of amino acids, *θ* is the degrees in elipticity, *l* is the path length and *c* is the concentration.

### Incucyte live-cell imaging

Cells were seeded onto 96-well tissue culture dishes at equal densities in six replicates. After attachment over-night, cells were transfected with MVI siRNA, or scrambled siRNA (Qiagen). Photomicrographs were taken every hour using an IncuCyte live-cell imager (Essen Biosciences, Ann Harbour, MI) and confluency of cultures was measured using IncuCyte software. Confluency values between wells were normalised to initial confluency for comparison.

### Chromatin accessibility assay

HeLa cell monolayers were cultured for 24 h before treatment with 25 µM TIP (Sigma), or DMSO control, for 1 h at 37 °C. Cells were trypsinized and washed 1× in PBS. Chromatin was then isolated, digested and then DNA purified using the Chromatin Accessibility Assay Kit (Abcam ab185901) according to manufacturer’s instructions. The samples were analysed by qPCR using primers in Table S3.

### RNA extraction and RT-qPCR

RNA from HeLa cells was extracted using Gene Jet RNA purification kit (Thermo scientific) according to manufacturer’s protocol. The RNA concentration was measured using Geneflow Nanophotometer and RT-qPCR was performed with one-step QuantiFast SYBR Green qPCR kit (Qiagen) using 50 ng of RNA in each sample. A list of qPCR primers is given in Supplementary Table 3.

### RNA-seq and analysis

Total RNA was extracted from three replicates of WT, MVI KD and Scrambled siRNA. Ice cold TRIzol reagent was added to each culture and homogenised. The mixture was then incubated for 5 min at room temperature then chloroform was added to the lysis and incubated for 3 min. The samples were then centrifuged at 12,000 × *g* at 4 °C. The colourless aqueous phase was collected. The RNA was then precipitated with incubation for 10 mins with isopropanol before centrifugation for a further 10 min at 12,000 × *g* at 4 °C. The pellet was washed in 75% (v/v) ethanol, vortexed and centrifuged for 5 min at 7500 × *g* at 4 °C. The RNA pellet is air dried for 10 min. The pellet is then resuspended in 50 µL of RNase-free water containing 0.1 mM EDTA and incubated at 55 °C for 15 min to allow the RNA to dissolve. The RNA was then quantified using then 260 nm absorbance, ensuring the A260/A280 ratio was ~2, therefore implying the sample is pure. The sample was then further purified using the RNeasy kit (Qiagen) where the manufacturers protocol was followed exactly. Once the purity and stability had been measured the RNA was then stored at −80 °C.

The RNA-seq libraries were prepared with TruSeq RNA Library Prep kit v2 as per protocol instructions. Resulting libraries concentration, size distribution and quality were assessed on a Qubit fluorometer with a dsDNA high sensitivity kit and on an Agilent 2100 bioanalyzer using a DNA 7500 kit. Then libraries were normalised, pooled and quantified with a KAPA Library quantification kit for Illumina platforms on a ABI StepOnePlus qPCR machine, then loaded on a high output flow cell and paired-end sequenced (2 × 75 bp) on an Illumina NextSeq 550 next generation sequencer (performed at the NYUAD Sequencing Centre). The raw FASTQ reads were quality trimmed using Trimmomatic (version 0.36)^57^ to trim low quality bases, systematic base calling errors, as well sequencing adapter contamination. FastQC (www.bioinformatics.babraham.ac.uk/projects/fastqc) was used to assess the quality of the sequenced reads pre/post quality trimming. Only the reads that passed quality trimming in pairs were retained for downstream analysis. The quality trimmed RNA-seq reads were aligned to the Homo sapiens GRch38.p4 genome using HISAT2 (version 2.0.4)^58^. The resulting SAM alignment files were then converted to BAM format and sorted by coordinate using SAMtools (version 0.1.19)^59^. The BAM alignment files were processed using HTseq-count^60^ using the reference annotation file to produce raw counts for each sample. The raw counts were then analyzed using the online analysis portal NASQAR (http://nasqar.abudhabi.nyu.edu/) in order to merge, normalise and identify differentially-expressed genes by using the START app^61^. Differentially-expressed genes by at least 2-fold log2(FC) ≥ 1 and adjusted *p* value of <0.05 for upregulated genes and log2(FC) ≤ −1 and adjusted *p* value of <0.05 for down-regulated genes) between the samples which were then subjected to Gene Ontology (GO) enrichment using ShinyGo v0.60 (http://bioinformatics.sdstate.edu/go/)^62^. RNA-Seq data were deposited in the Gene Expression Omnibus (GEO) database under the accession number GSE149448.

### Chromatin immunoprecipitation (ChIP)

To identify specific RNAPII-DNA interactions, ChIP was performed using mouse anti-RNAPII-pSer5 antibody (Abcam Ab5408). A confluent T175 flask (10 × 10^6^–30 × 10^6^) of HeLa cells was crosslinked by adding formaldehyde dropwise directly to the media to a final concentration of 0.75% and was left for gentle rotation at room temperature (RT) for 10 min. To stop the reaction, glycine was added to a final concentration of 125 mM and was incubated with shaking for 5 min at RT. The cells were washed twice with 10 ml of cold PBS and were scraped in 5–8 ml of cold PBS. All cells were collected and centrifuged at 1000 × *g*, 4 °C for 5 min. The pellet was resuspended in ChIP lysis buffer (750 μL per 1 × 10^7^ cells) (lysis buffer: 50 mM HEPES-KOH pH 7.5, 140 mM NaCl, 1 mM EDTA pH8, 1% Triton X-100, 0.1% Sodium Deoxycholate, 0.1% SDS and Protease Inhibitors) and was incubated on ice for 10 min. The cells were sonicated using the diagenode bioruptor sonicator in order to shear DNA to an average fragment size of 200-800 bp. The fragment size was analysed on a 1.5% agarose gel. After sonication, cell debris was removed by centrifugation for 10 min, 4 °C, 8000 × *g* and the supernatant (chromatin) was used for the immunoprecipitation. The sonicated chromatin was snap frozen on dry ice and was stored at −80 °C until further use (max storage 3 months).

The chromatin prepared above was diluted 1:10 with RIPA buffer (50 mM Tris-HCl pH8, 150 mM NaCl, 2 mM EDTA pH8, 1% NP-40, 0.5% Sodium Deoxycholate, 0.1% SDS and Protease Inhibitors) and was distributed into 6 tubes (~1 × 10^6^ cells per IP)—three samples for specific antibody (MVI) and three samples for the no antibody control (beads only). 10% of diluted chromatin was removed to serve as input sample and was stored at −20 °C until further use. All chromatin samples were pre-cleared using the protein A magnetic beads (Thermo Fisher Scientific) for 30 min after which 20 µl of mouse anti-RNAPII-pSer5 antibody was added to each of the triplicate Ab samples (1 in 50 dilution) and the tubes were rotated at 4 °C, over-night. Next day, 40 µl of protein A magnetic beads (washed three times in RIPA buffer) were added to each of the samples including the no antibody control tubes and were put on rotation at 4 °C for 1 h. After 1 h, the beads were collected using a magnetic rack and were washed twice in low salt buffer (0.1% SDS, 1% Triton X-100, 2 mM EDTA, 20 mM Tris-HCl pH 8, 150 mM NaCl), once in high salt buffer (0.1% SDS, 1% Triton X-100, 2 mM EDTA, 20 mM Tris-HCl pH 8, 500 mM NaCl), once in LiCl wash buffer (0.25 M LiCl, 1% NP-40, 1% Sodium Deoxycholate, 1 mM EDTA, 10 mM Tris-HCl pH 8) and finally in TE (10 mM Tris pH8, 1 mM EDTA). DNA was eluted by adding 120 µl of elution buffer (1% SDS, 100 mM NaHCO_3_) to the beads and vortexing them slowly for 15 min at 30 °C. To reverse crosslink the protein-DNA complexes, 4.8 µl of 5 M NaCL and 2 µl RNase A (10 mg/ml) was added to the elutes including the input sample that was stored at −20 °C and they were incubated while shaking at 65 °C over-night followed by proteinase K treatment at 60 °C for 1 h. The DNA was then purified using phenol:chloroform extraction and the samples were analysed by qPCR using primers in Supplementary Table 3.

### Steady-state ATPase activity

Ca^2+^-actin monomers were converted to Mg^2+^-actin with 0.2 mM EGTA and 50 μM MgCl_2_ before polymerising by dialysis into 20 mM Tris.HCl (pH7.5), 20 mM imidazole (pH 7.4), 25 mM NaCl and 1 mM DTT. A 1.1 molar equivalent of phalloidin (Sigma) was used to stabilise actin filaments^63,64^.

Steady-state ATPase activities were measured at 25 °C in KMg50 buffer (50 mM KCl, 1 mM MgCl_2_, 1 mM EGTA, 1 mM DTT, and 10 mM imidazole, pH 7.0). Supplemented with the NADH-coupled assay components, 0.2 mM NADH, 2 mM phosphoenolpyruvate, 3.3 U ml^−1^ lactate dehydrogenase, 2.3 U ml^−1^ pyruvate kinase and various actin concentrations (0–30 μM). The final [Mg.ATP] was 5 mM and MVI concentration was 100–300 nM. The assay was started by the addition of MVI. The change in absorption at OD_340_ nm was followed for 5 min. The *k*_cat_ and *K*_actin_ values were determined by fitting the data to Eq. (2).

2 $${Rate}\,=\,{V}_{{{{{{\rm{o}}}}}}}\,+\,\,\left(\frac{{k}_{{{{{{\rm{cat}}}}}}}[{Actin}]}{{K}_{{{{{{\rm{actin}}}}}}}\,+\,[{Actin}]}\right)$$

*V*_o_ is the basal ATPase activity of MVI, *k*_cat_ is the maximum actin-activated ATPase rate and *K*_actin_ is the concentration of actin needed to reach half maximal ATPase activity.

### Graphics

Unless stated, data fitting and plotting was performed using Plots of data^65^ and Grafit Version 5 (Erithacus Software Ltd). Cartoons were generated using the BioRender software.

### Statistics and reproducibility

All experiments were performed from a minimum of three independent experiments.

### Reporting summary

Further information on research design is available in the Nature Research Reporting Summary linked to this article.

## Supplementary information

Supplementary Information

Description for Additional Supplementary Files

Supplementary Movie 1

Supplementary Movie 2

Supplementary Movie 3

Supplementary Movie 4

Supplementary Movie 5

Supplementary Movie 6

Supplementary Movie 7

Supplementary Movie 8

Supplementary Movie 9

Supplementary Movie 10

Supplementary Movie 11

Reporting Summary

## Data Availability

The data that support this study are available from the corresponding author upon reasonable request. RNA-seq data generated in this study have been deposited in the Gene Expression Omnibus (GEO) database under the accession number GSE149448. Source data are provided with this paper.

## Source data

Source Data
