# Supplementary material for: Myosin VI regulates the spatial organisation of mammalian transcription initiation
Source: Nat Commun. 2022 Mar 15;13:1346. doi: 10.1038/s41467-022-28962-w (PMC8924246; doi:10.1038/s41467-022-28962-w)
Supplement: Supplementary file 1 — Supplementary Information [file 41467_2022_28962_MOESM1_ESM.pdf]

SUPPLEMENTARY INFORMATION

Myosin VI regulates the spatial organization of mammalian transcription initiation.

Yukti Hari-Gupta, Natalia Fili, Ália dos Santos, Alexander W. Cook, Rosemarie E. Gough, Hannah C. W. Reed, Lin Wang, Jesse Aaron, Tomas Venit, Eric Wait, Andreas Grosse-Berkenbusch, J. Christof M. Gebhardt, Piergiorgio Percipalle, Teng-Leong Chew, Marisa Martin-Fernandez and Christopher P. Toseland

A

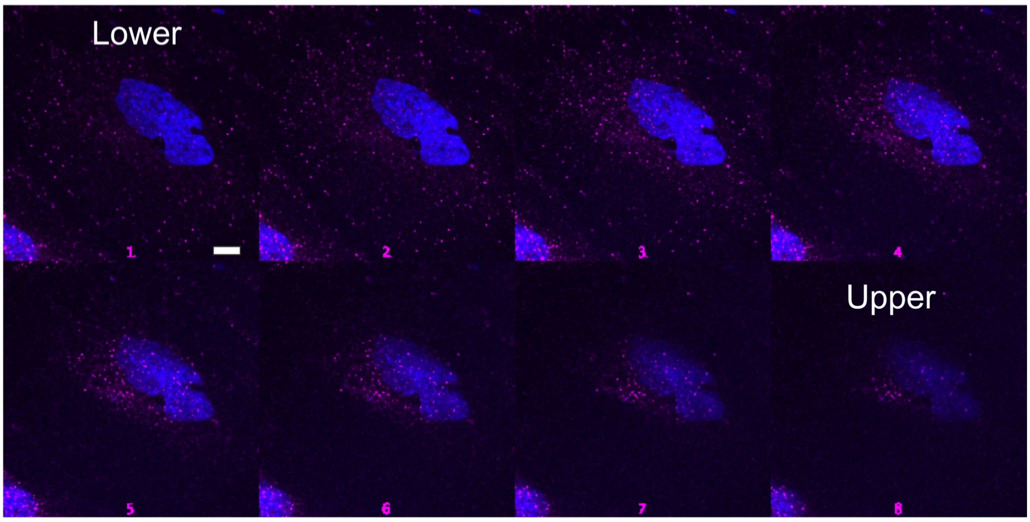

B

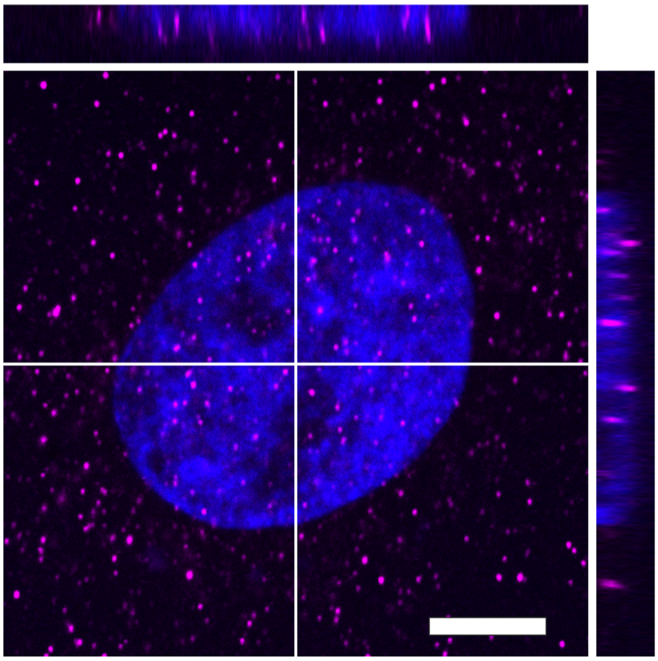

Supplementary Fig. 1 Nuclear localisation of MVI. (A) Immunofluorescence staining against MVI (magenta) and DNA (cyan) in HeLa cells. Images where acquired at 400 nm intervals in the z axis through the nuclear body. (scale bar 5 μm). (B) A cross-section is presented depicting MVI in the nucleus. (scale bar 5 μm).

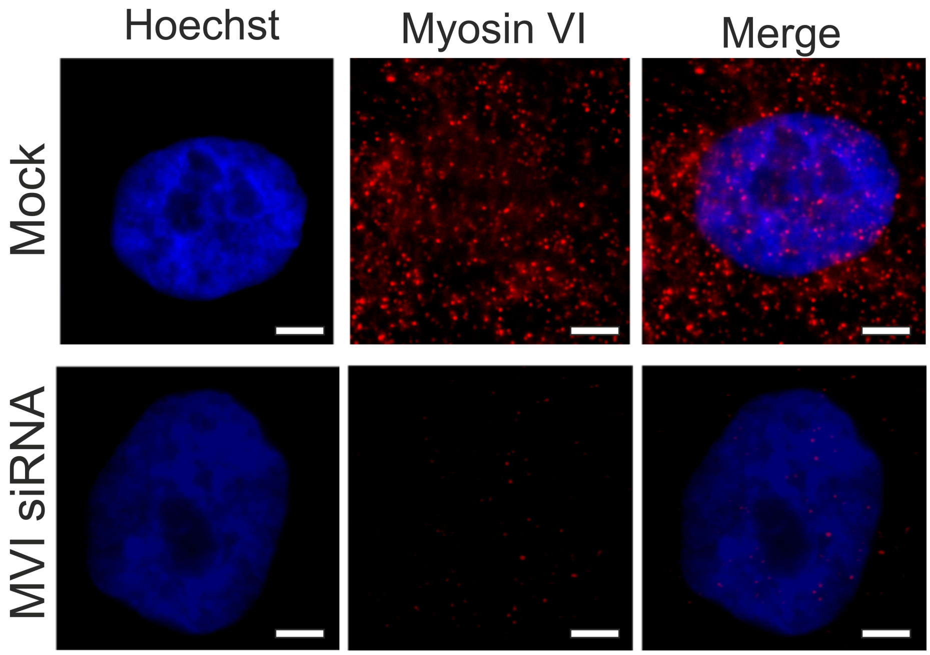

Supplementary Fig. 2 Representative images of myosin VI following siRNA knockdown. Widefield Immunofluorescence staining against myosin VI (MVI) (magenta) and DNA (cyan) in HeLa cells under mock (control siRNA treatment) and siRNA conditions following 48 hr transfection. Images were acquired at the mid-point of the nucleus (scale bar 5 μm).

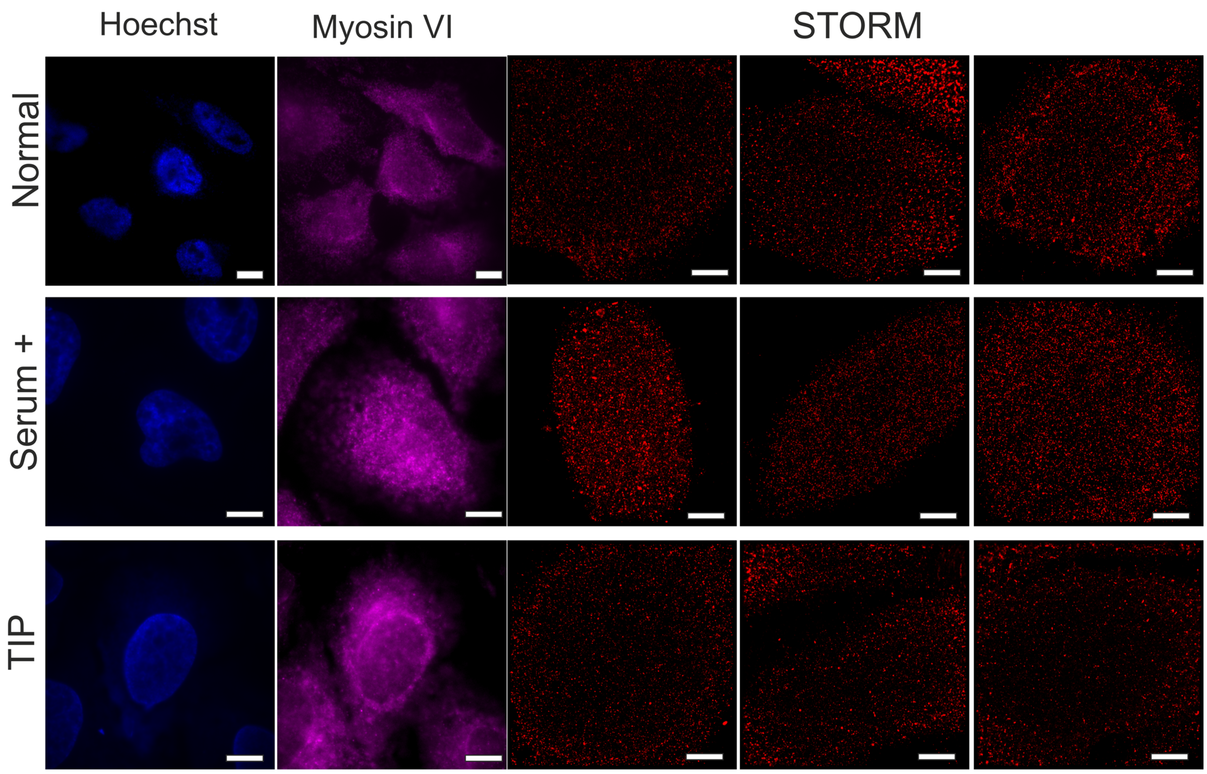

Supplementary Fig. 3 Further examples of MVI nuclear organisation. (Left) Example confocal images following immunofluorescence staining against MVI (magenta) and DNA (cyan) in HeLa cells under normal, serum stimulated and TIP-treated conditions (Scale bar 5 μm). (Right) Example STORM render images of MVI under normal, serum and TIP-treated conditions, as described in the Methods (scale bar 2 μm). Z-stack of Normal condition is shown in Supplementary Movie 1 and serum treated condition is shown in Supplementary Movie 4.

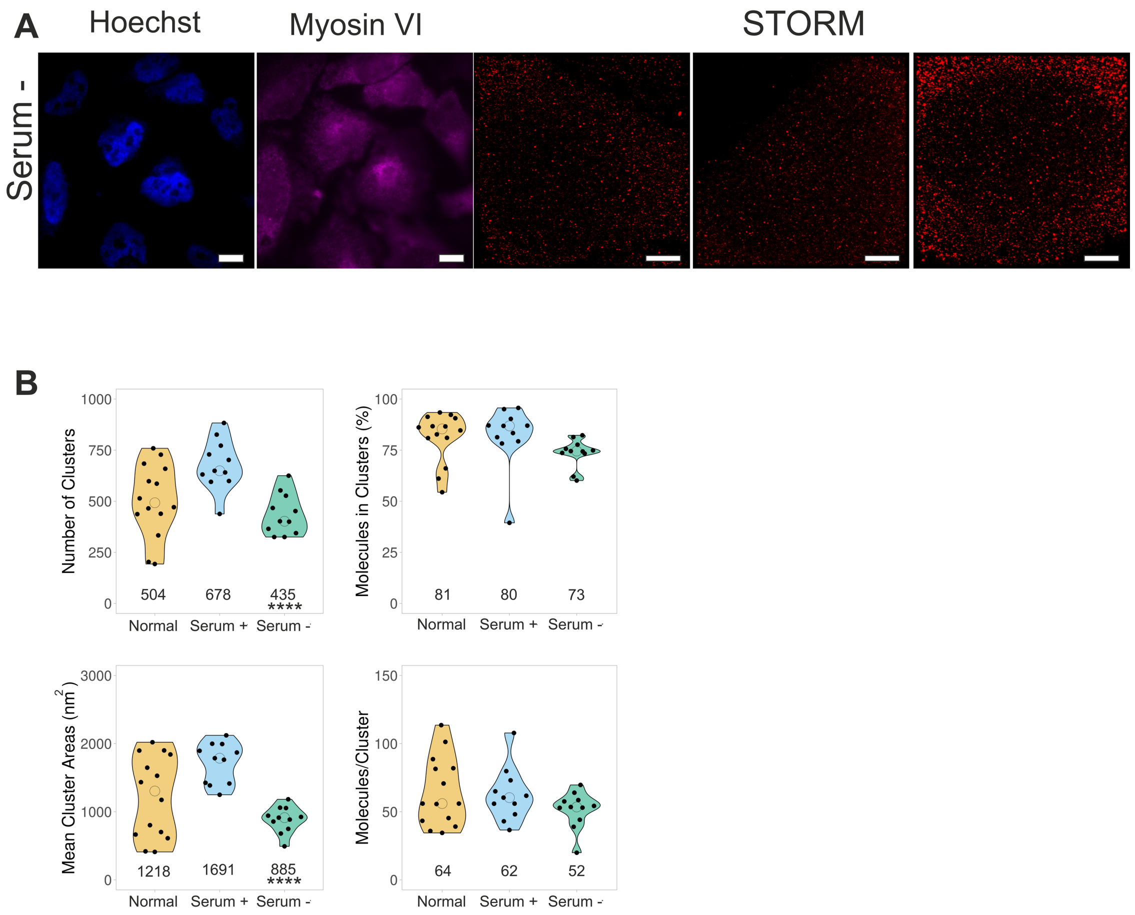

Supplementary Fig. 4 MVI nuclear organisation following serum-starvation. (A) Example confocal (Scale bar 10µm) and STORM render (Scale bar 2µm) of MVI following immunofluorescence staining. Z-stack is shown in Supplementary Movie 5. (B) Cluster analysis of MVI nuclear organisation under normal, serum-treated and serum-starved conditions. Individual data points correspond to the average value for a cell ROI (n = 14 for normal, 11 for serum-treated and 11 for serum-starved). The values represent the mean from the ROIs for each condition (Only statistically significant changes are highlighted *p <0.05, **p <0.01 by two-tailed t-test comparing serum-stimulated to starved conditions).

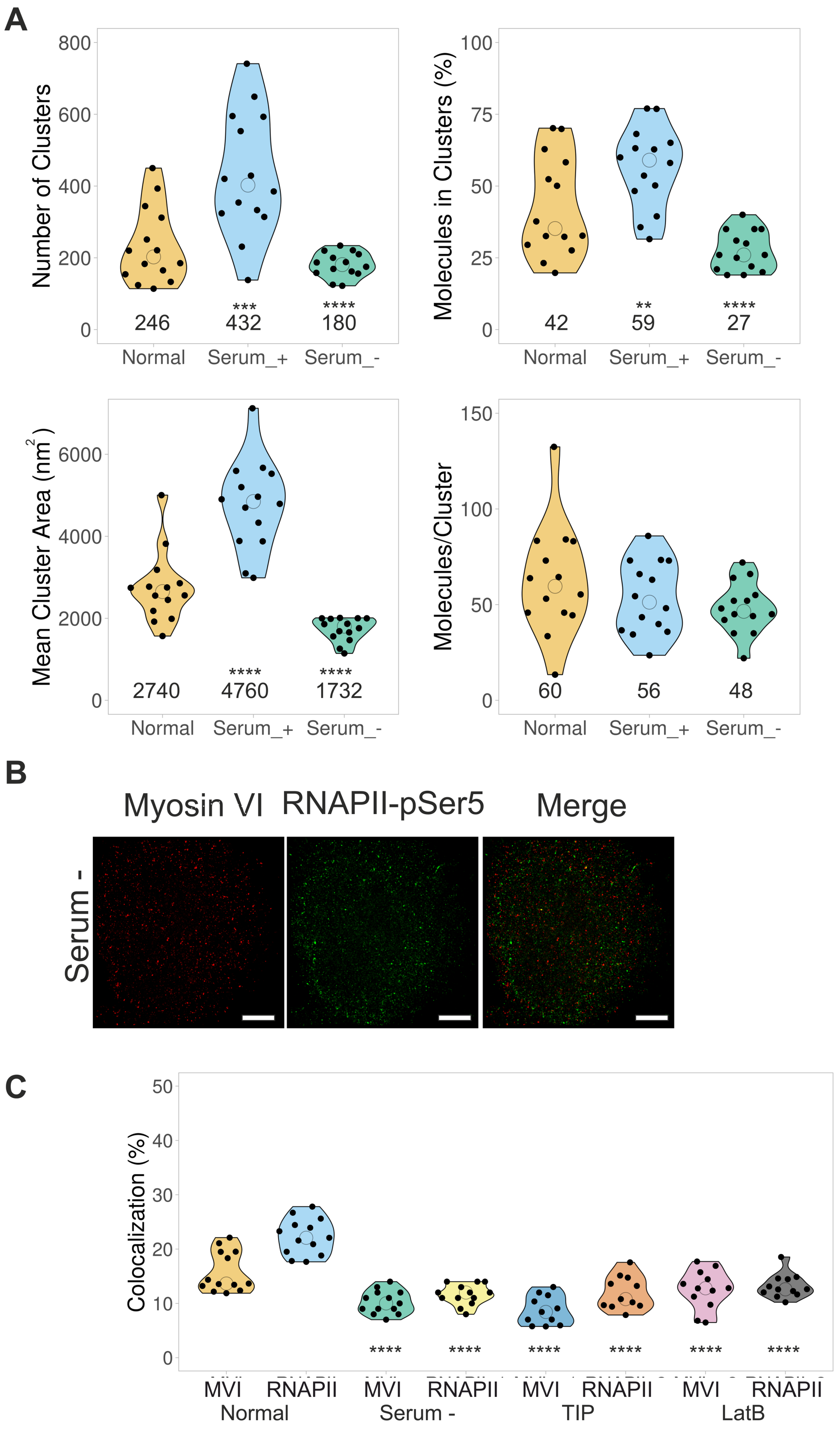

Supplementary Fig. 5 Cluster analysis and colocalization of MVI and RNAPII-pSer5. (A) Cluster analysis of RNAPII nuclear organisation under normal, serum-treated and starved conditions. Individual data points correspond to the average value for a cell ROI (n=14). The values represent the mean from the ROIs for each condition. (Only statistically significant changes are highlighted **p <0.01, ***p <0.001, ****p <0.0001 by two-tailed t-test compared to normal conditions). (B) Example STORM render image of MVI and RNAPII-pSer5 under serum-starvation conditions (scale bar 2 μm). (C) Colocalization analysis of myosin VI (MVI) and RNAPII-pSer5 clusters under normal (n=13), serum-starvation (n=13), TIP- (n=11) and

LatB-treated (n=11) conditions. Individual data points represent the percentage of each protein which is colocalized based on a DoC value above 0.4. (Only statistically significant changes are highlighted ****p <0.0001 by two-tailed t-test compared to normal conditions for each protein).

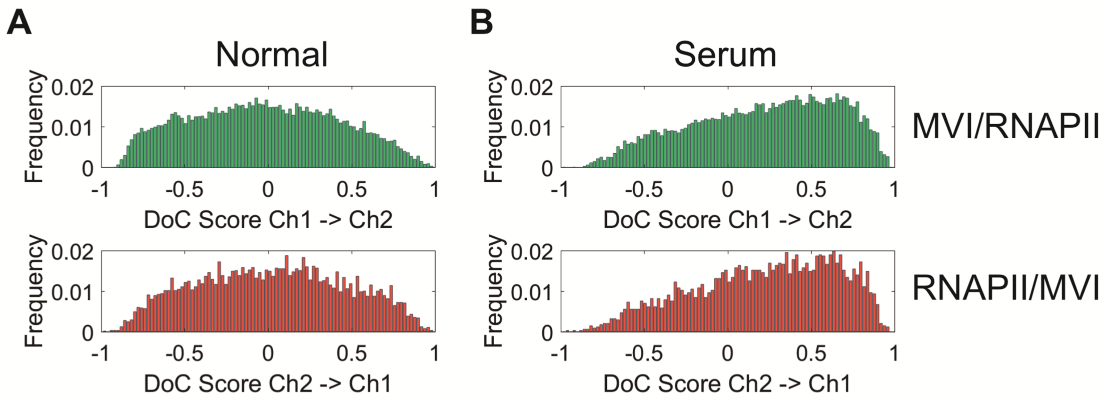

Supplementary Fig. 6 Colocalisation of MVI and RNAPII. Representative DoC value histograms for MVI colocalised with RNAPII and RNAPII colocalised with MVI under normal (A) and Serum stimulated conditions (B). The DoC values are calculated using coordinate-based colocalization analysis (26) in Clus-DoC (21). -1 is segregated, 0 is random distribution and +1 is colocalised. A threshold is applied at values about +0.4 to determine colocalised molecules, as described in (21).

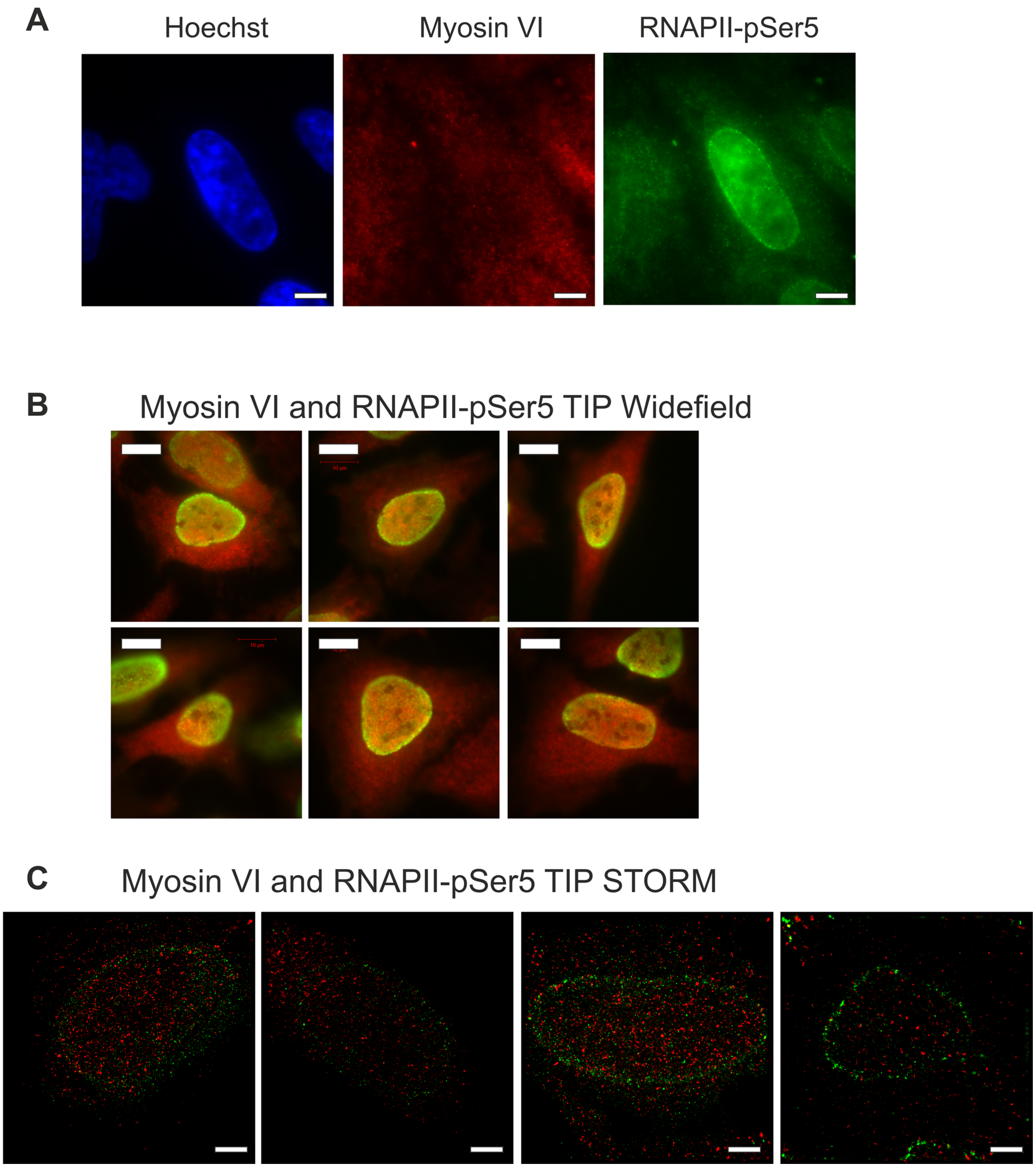

Supplementary Fig. 7 Representative images of myosin VI and RNAPII under TIP treatment. (A) Example immunofluorescence image of Myosin VI and RNAPII-pSer5 following TIP-treatment (scale bar 5 μm). Z-stack is shown in Supplementary Movie 6. (B) Further example images of myosin VI (red) and RNAPII-pSer5 (green) in HeLa cells when treated with TIP. Images were acquired at the mid-point of the nucleus (scale bar 10 μm). (C) Further example STORM renders of myosin VI and RNAPII-pSer5 (scale bar 2 μm).

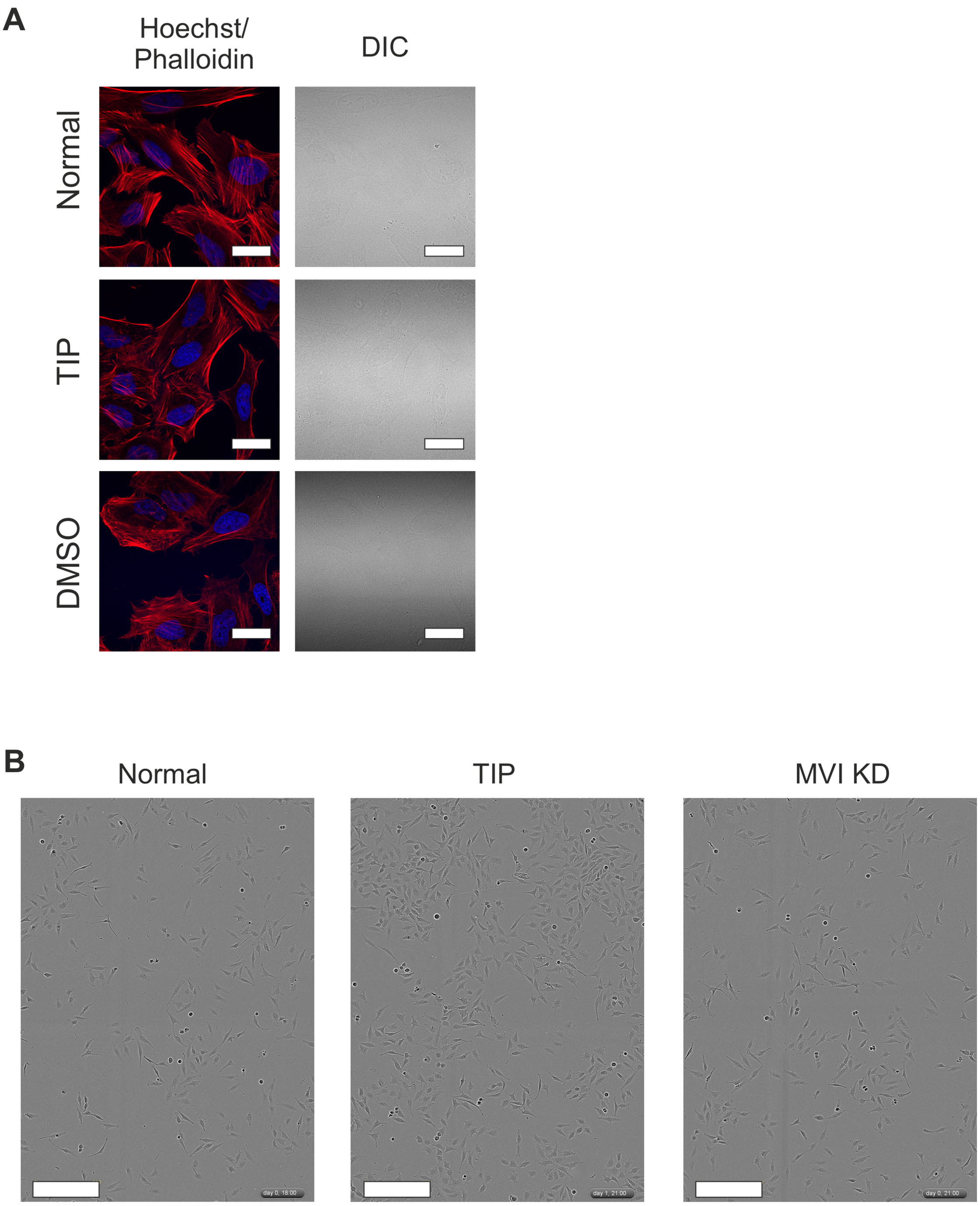

Supplementary Fig. 8 Impact of MVI perturbation on the cytoskeleton and cell shape. (A) Staining of HeLa cells with Hoechst and Rhodamine phalloidin to stain nuclei and actin cytoskeleton. Experiments were performed under normal, TIP-treated and carrier control conditions. The corresponding DIC image is also shown (scale bar 10 μm). (B) High content transmission images of live HeLa cells growing under normal, TIP treated and MVI knockdown conditions (scale bar 300 μm).

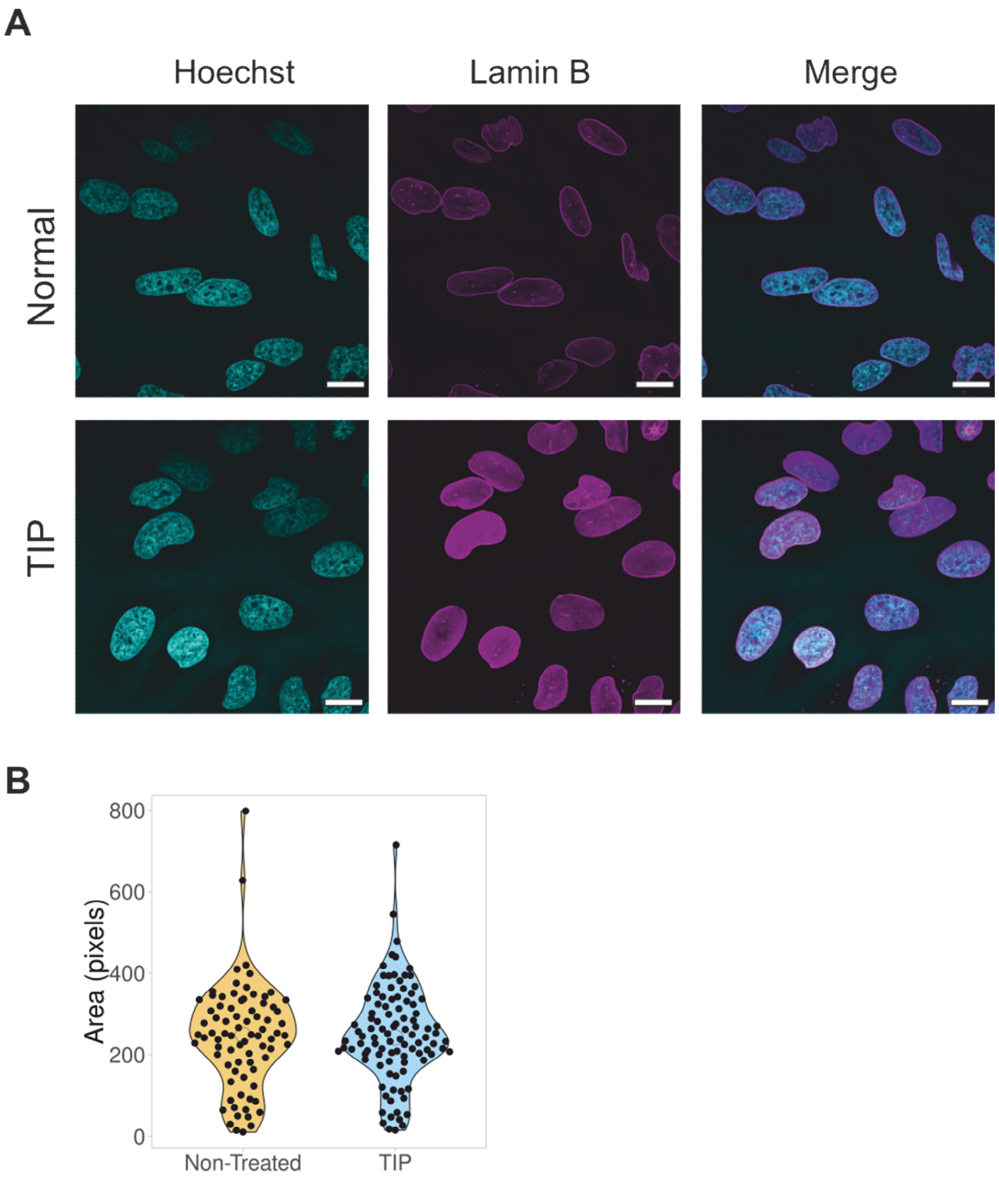

Supplementary Fig. 9 Impact of TIP treatment on nuclear integrity and size. (A) Representative images of lamin B immunofluorescence staining following under normal growth and TIP treatment conditions (Scale bar 15 μm). (B) Quantification of nuclear area under conditions used in (A).

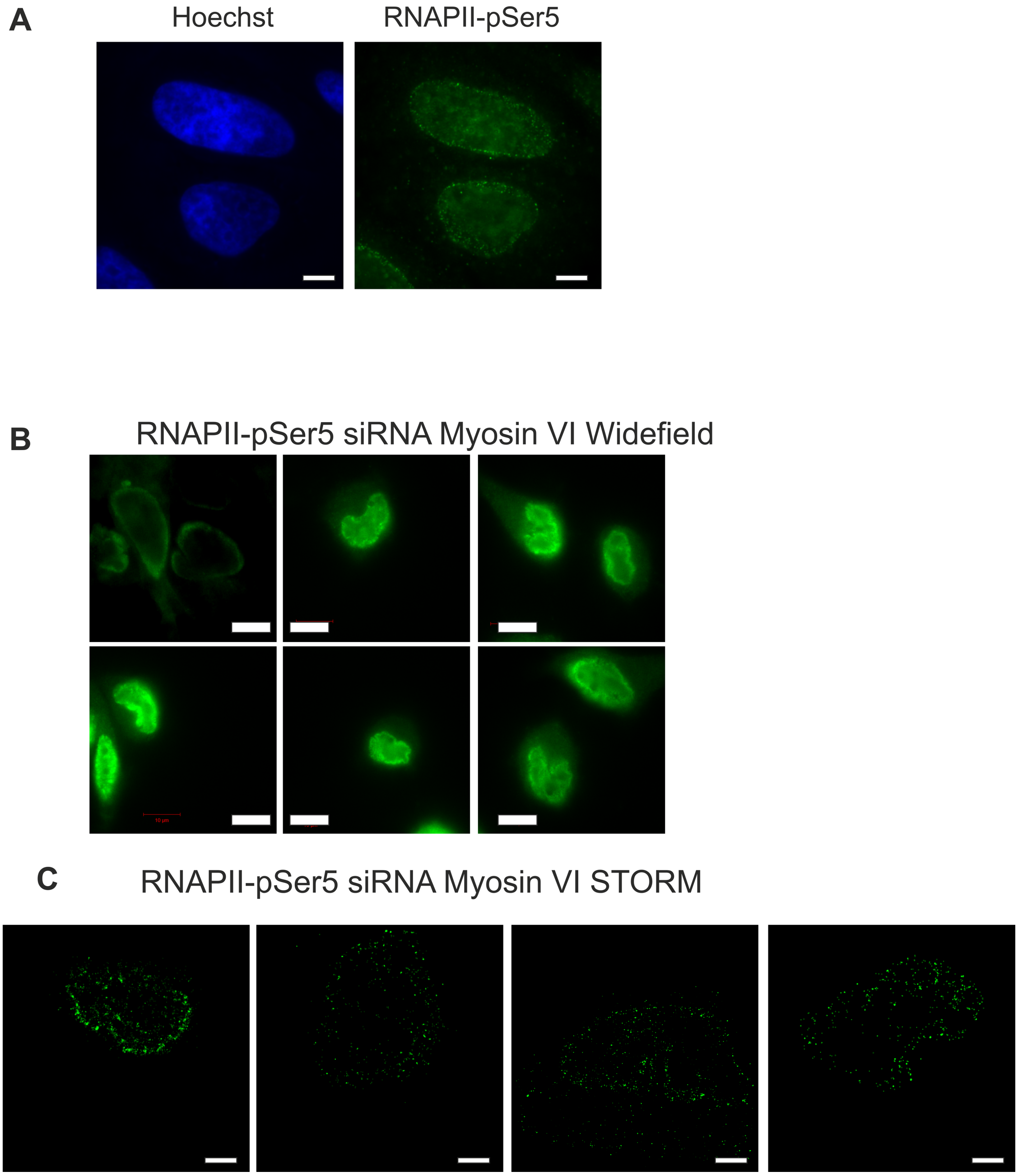

Supplementary Fig. 10 Representative images of RNAPII following Myosin VI siRNA knockdown. (A) Example image of RNAPII-pSer5 following immunofluorescence staining after myosin VI siRNA knockdown (scale bar 5 μm). Z-stack is shown in Supplementary Movie 7. (B) Further example images of RNAPII-pSer5 in HeLa cells following myosin VI knockdown. Images were acquired at the mid-point of the nucleus (scale bar 10 μm). (C) Further example STORM renders of RNAPII-pSer5 (scale bar 2 μm). Please see Supplementary Fig. 2 for myosin VI controls.

A

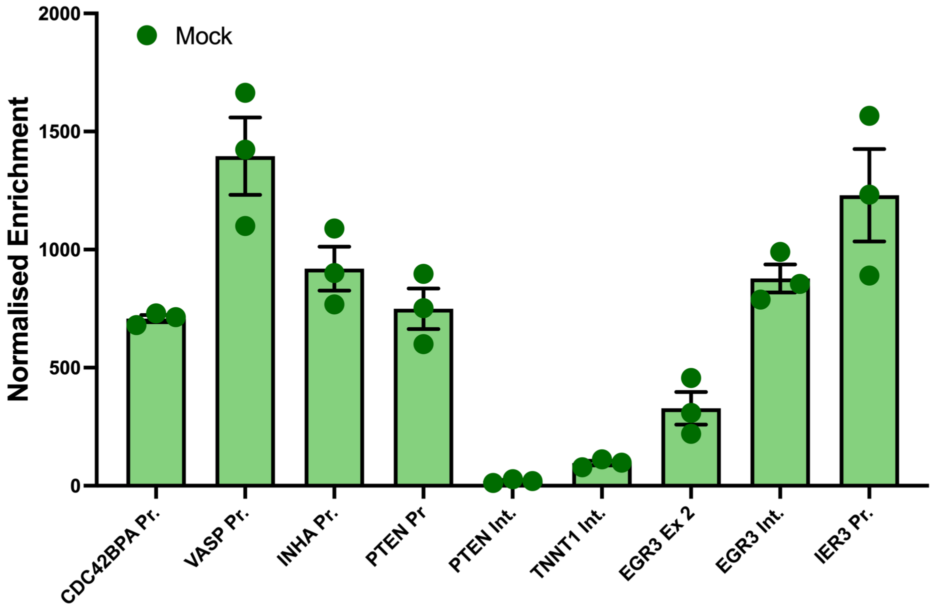

B

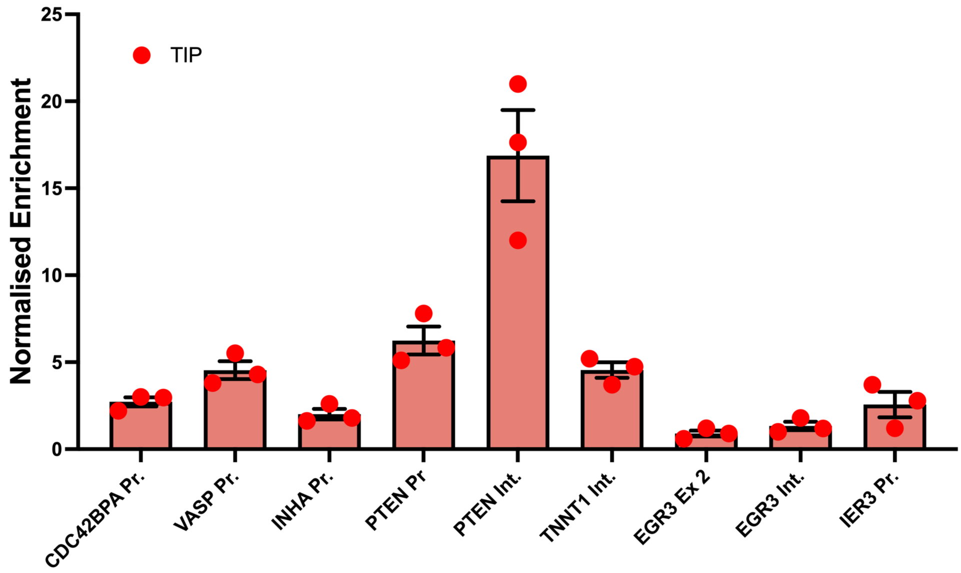

Supplementary Fig. 11 RNAPII-pSer5 chromatin immunoprecipitation. (A) RNAPII-pSer5 ChIP against labelled loci under normal and TIP-treated conditions. Values are the average of three independent experiments. Error bars represent SEM from three independent experiments. (B) RNAPII-pSer5 ChIP against labelled loci following TIP-treatment. Values are the average of three independent experiments. Error bars represent SEM from three independent experiments.

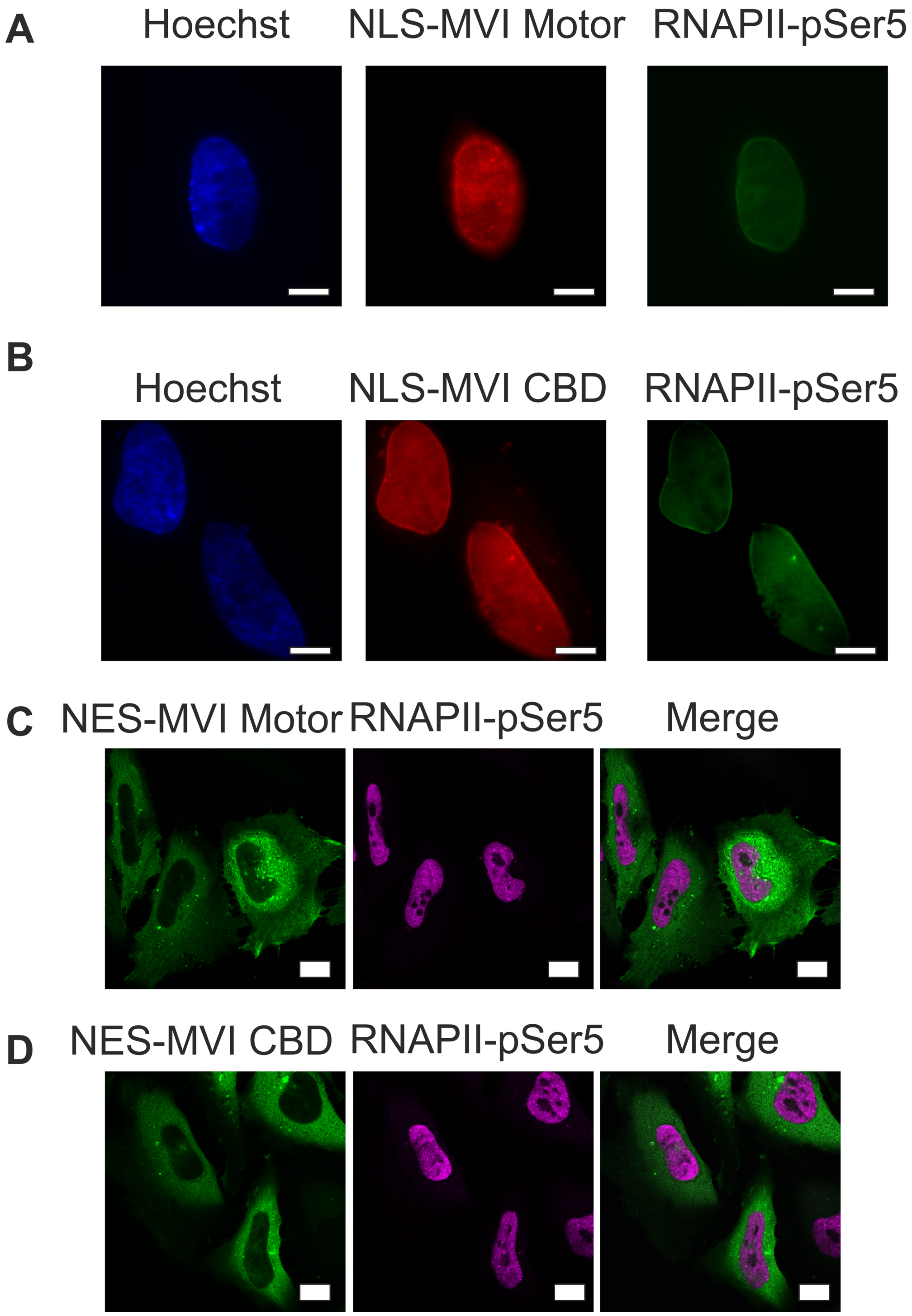

Supplementary Fig. 12 Representative images of NLS myosin VI motor and CBD with RNAPII. (A) Widefield Halo-TMR and Immunofluorescence staining against NLS myosin VI motor (red) and RNAPII-pSer5 (green) in HeLa cells. Images were acquired at the mid-point of the nucleus (scale bar 5 μm). Z-stack is shown in Supplementary Movie 8. (B) Widefield Halo-TMR and Immunofluorescence staining against NLS myosin VI CBD (red) and RNAPII-pSer5 (green) in HeLa cells. Z-stack is shown in Supplementary Movie 9. (C) Confocal imaging of NES-GFP-MVI-Motor (green) and immunofluorescence staining against RNAPII-pSER5 (red). (D) Confocal imaging of NES-GFP-MVI-CBD (green) and immunofluorescence staining against RNAPII-pSER5 (red). Images were acquired at the mid-point of the nucleus (scale bar 5 μm).

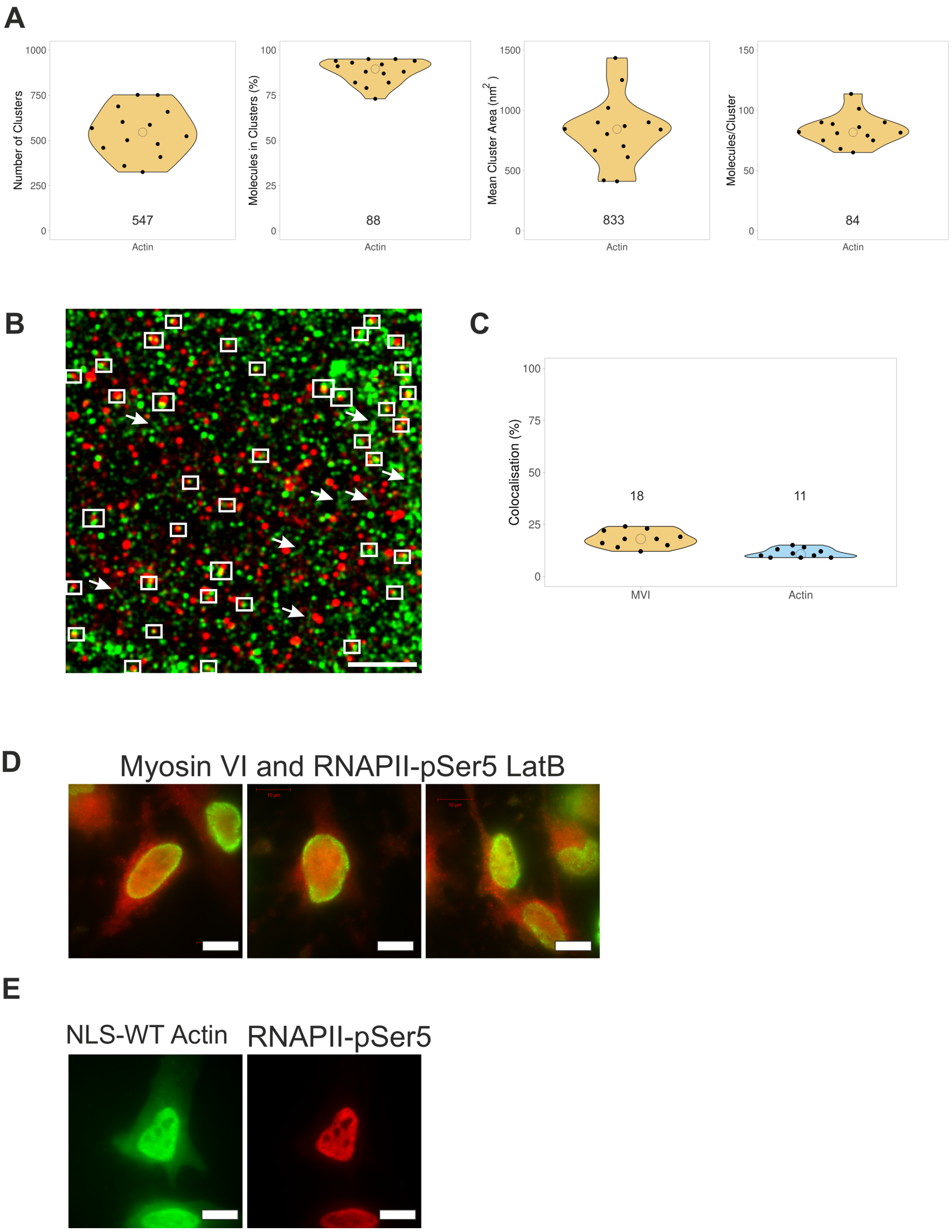

Supplementary Fig. 13 Perturbation of nuclear actin. (A) Cluster analysis of actin nuclear organisation. Individual data points correspond to the average value for a cell ROI (n=14). The values represent the mean from the ROIs for each condition. (B) Example STORM render of MVI (red) and actin (green) following immunofluorescence staining. Both proteins form clusters and those in close proximity are marked by a box. Arrows indicated short filamentous structures (scale bar 1 μm). (C) Colocalization analysis of myosin VI (MVI) and actin clusters (n=10). Individual data points represent the percentage of each protein which is colocalized based on a DoC value above 0.4. (D) Widefield Immunofluorescence staining against myosin VI (red) and RNAPII-pSer5 (green) in HeLa cells when treated with LatB. Images were acquired at the mid-point of the nucleus (scale bar 10 μm). (E) Example widefield image of WT YFP-NLS actin and immunofluorescence staining against RNAPII-pSer5 (scale bar 10 μm).

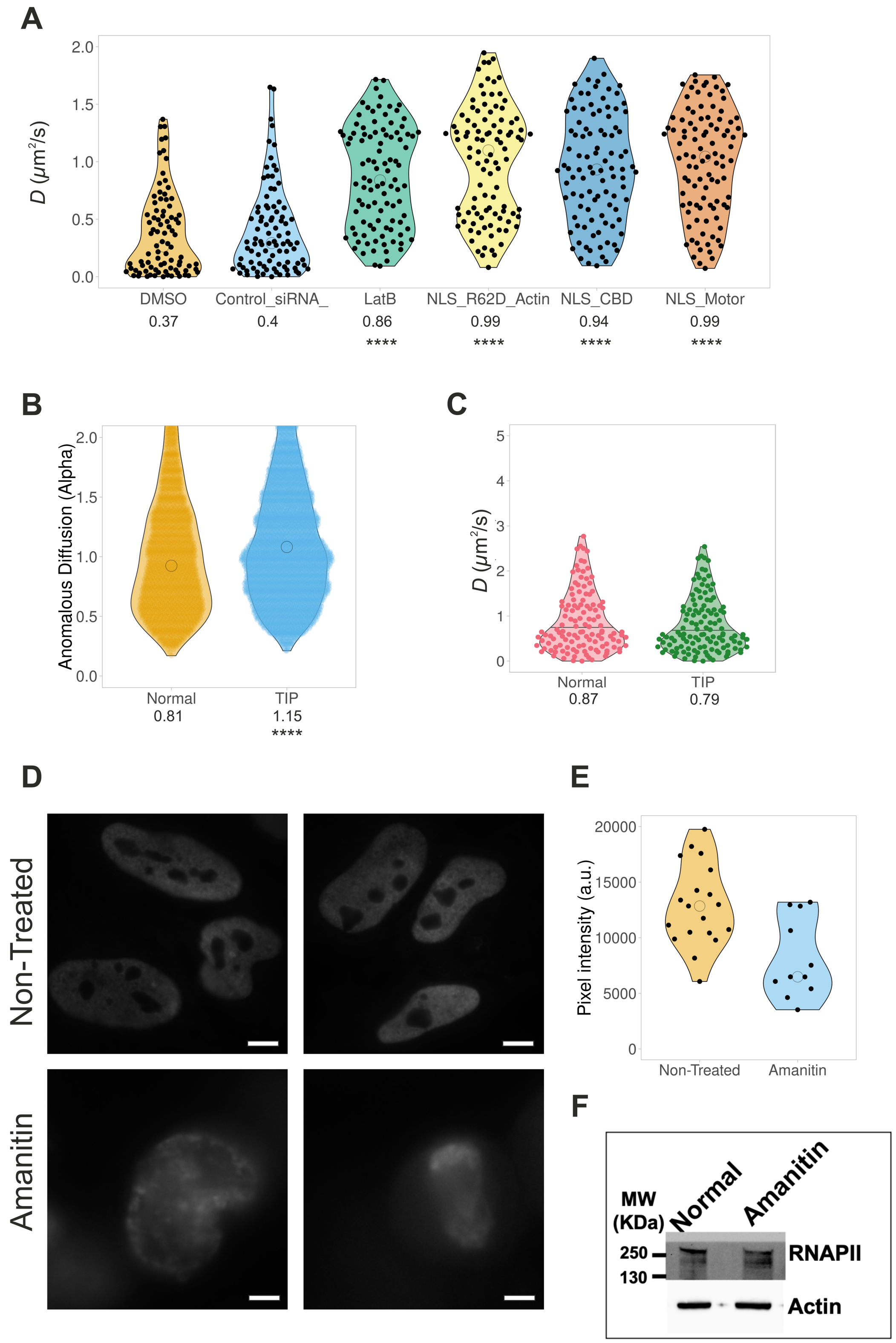

Supplementary Fig. 14 Controls for Live cell single molecule tracking. (A) Plot of Halo-RNAPII diffusion constants under the stated conditions derived from fitting trajectories to an anomalous diffusion model, as described in methods. Individual data points correspond to the average value for a cell ROI (n = 100). The values represent the mean from the ROIs for each condition (Only statistically significant changes are highlighted ****p <0.0001 by two-tailed t-test compared to normal conditions). NLS-R62D Actin, NLS CBD and NLS Motor refer to tracking of RNAPII following transfection of these constructs. NLS CBD and NLS motor were transfected in to Halo-RNAPII cells. (B) Plot of Halo-RNAPII anomalous diffusion alpha value

under normal and TIP treated conditions. Values were derived from fitting trajectories to an anomalous diffusion model, as described in methods. Data points correspond to the individual tracks across all cells (n = 100). The values represent the mean from the ROIs for each condition. (****p <0.0001 by two-tailed t-test compared to normal conditions). (C) Plot of SNAP-tag diffusion constants under the stated conditions derived from fitting trajectories to an anomalous diffusion model, as described in methods. Data points correspond to the average value for a cell ROI (n=100). The values represent the mean from the ROIs for each condition. There is not a statistically significant change between the datasets. (D) Example immunofluorescence staining against RNAPII-pSer5 under non-treated and amanitin treated conditions (scale bar 5 μm). (E) Quantification of mean nuclear pixel intensity for immunofluorescence staining against RNAPII-pSer5. (F) Corresponding western-blot against RNAPII-pSer5 under non-treated (Normal) and amanitin treated conditions.

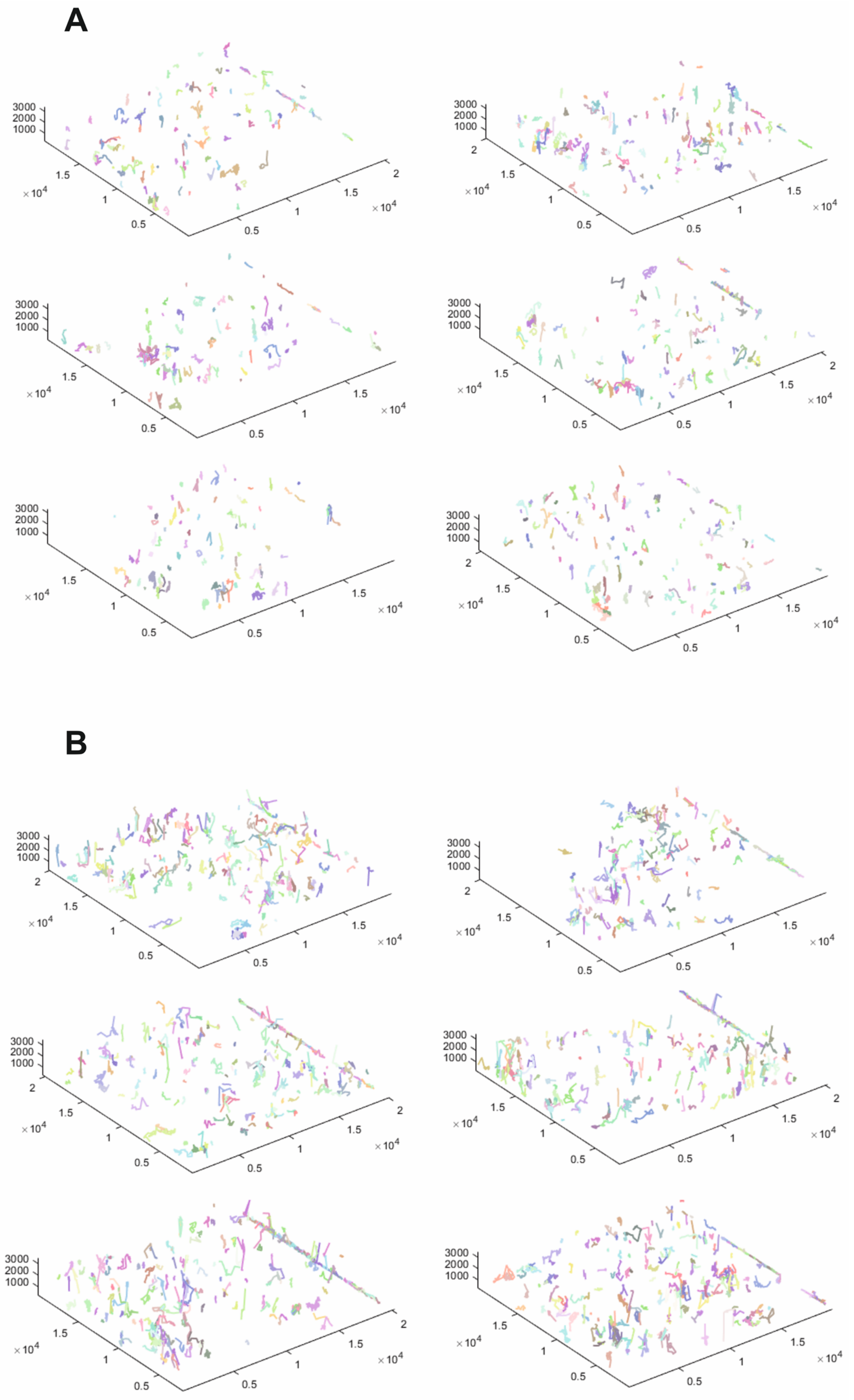

Supplementary Fig. 15 3D single molecule tracking examples. 3D single molecule trajectories under (A) normal and (B) TIP-treated conditions.

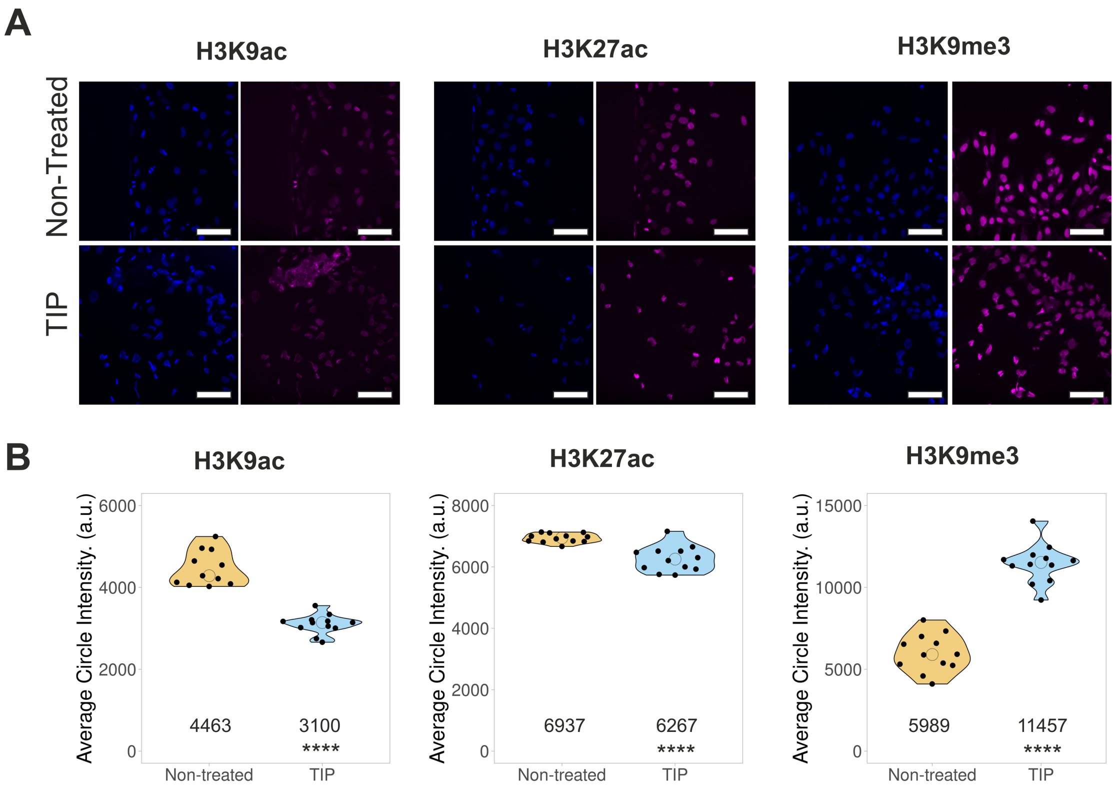

Supplementary Fig. 16 High-content screening of histone marks. (A) Example immunofluorescence staining against stated histones (magenta) and DNA (blue) in HeLa cells under normal and TIP-treated conditions (scale bar 10 μm). (B) The fluorescence intensity within the nucleus was measured for each histone marker under untreated and TIP-treated conditions, and each data point represents the average from a minimum of 1000 nuclei (Only statistically significant changes are highlighted ****p <0.0001 by two-tailed t-test compared to untreated conditions).

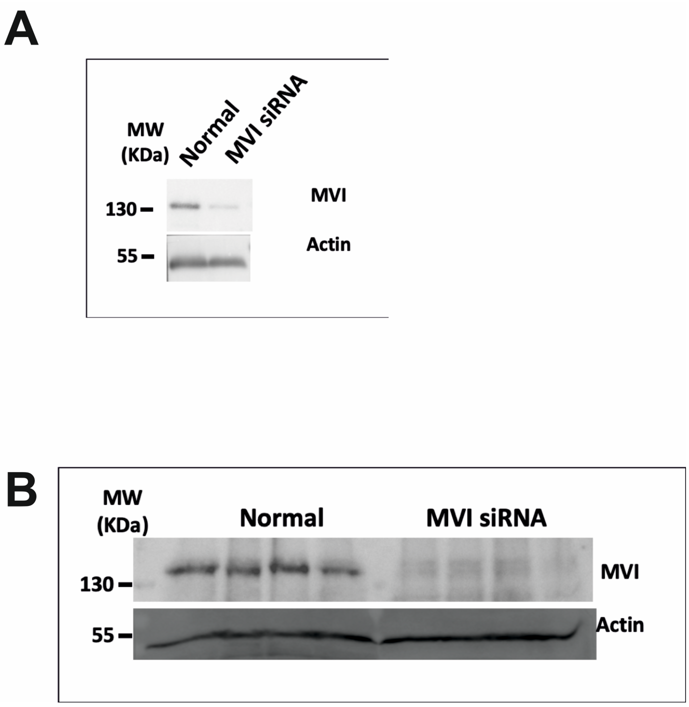

Supplementary Fig. 17 Western-blots corresponding to sample used in Figure 6 and 7. (A). Western-blot against MVI under normal and MVI-knockdown (MVI siRNA) conditions used in (Figure 6C) shows a 97% reduction in MVI. (B) Western-blot against MVI under normal and MVI-knockdown conditions. Samples were used for the RNA-seq analysis and serum-stimulation experiments in Figure 7.

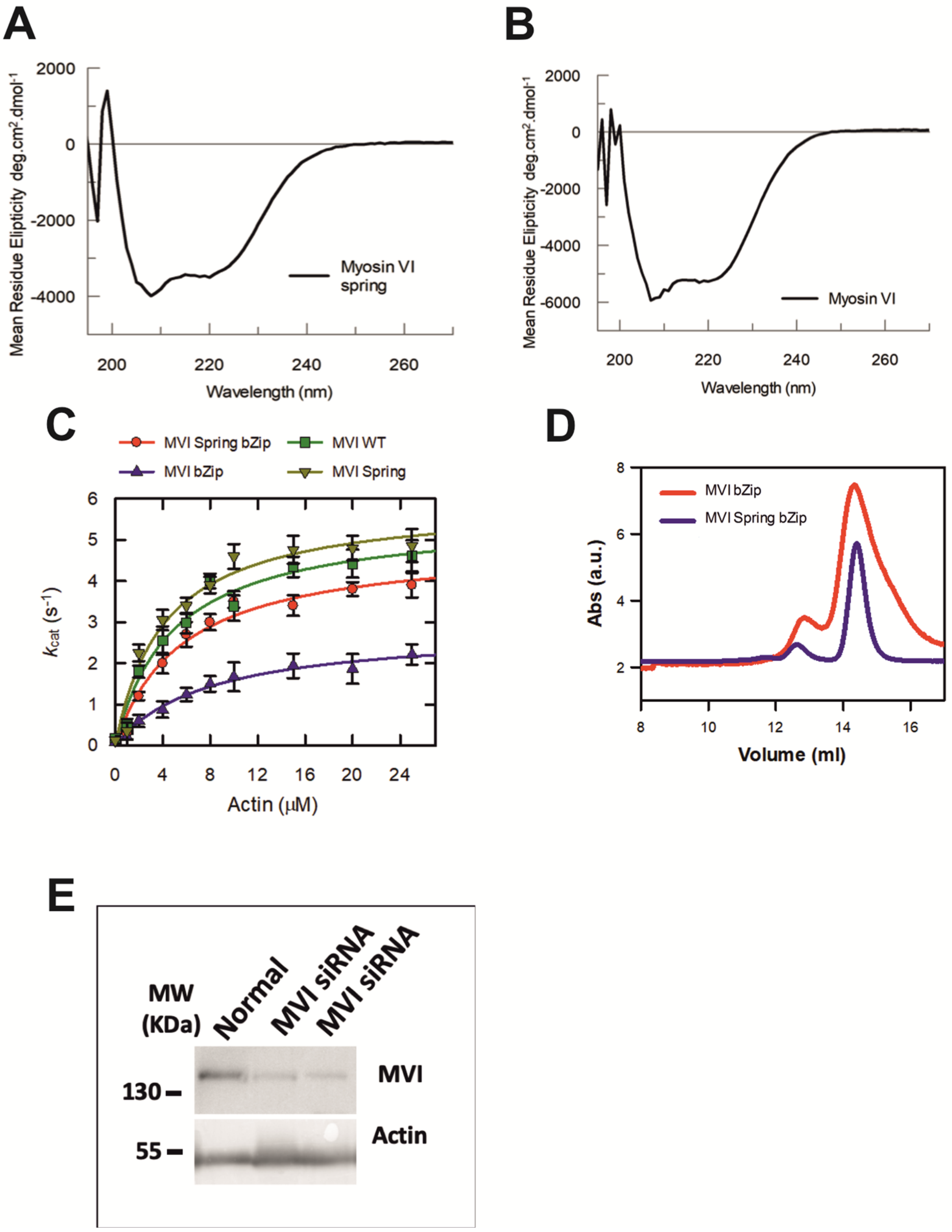

Supplementary Fig. 18 Characterisation of myosin VI spring construct. (A) Representative CD spectra for recombinant full-length myosin VI spring. (B) Representative CD spectra for recombinant full-length wild type myosin VI. (C) Michaelis-Menten plot of displaying steady-state actin-activated ATPase activity for the myosin VI (MVI) constructs. Error bars represent SEM from three-independent experiments. (D) Representative size exclusion chromatography trace for 1 mg/ml myosin VI bZip and myosin VI spring bZip constructs. (E) Western-blot against MVI under normal and MVI knockdown conditions before transfection with Halo-MVI(WT) and Halo-MVI Spring.

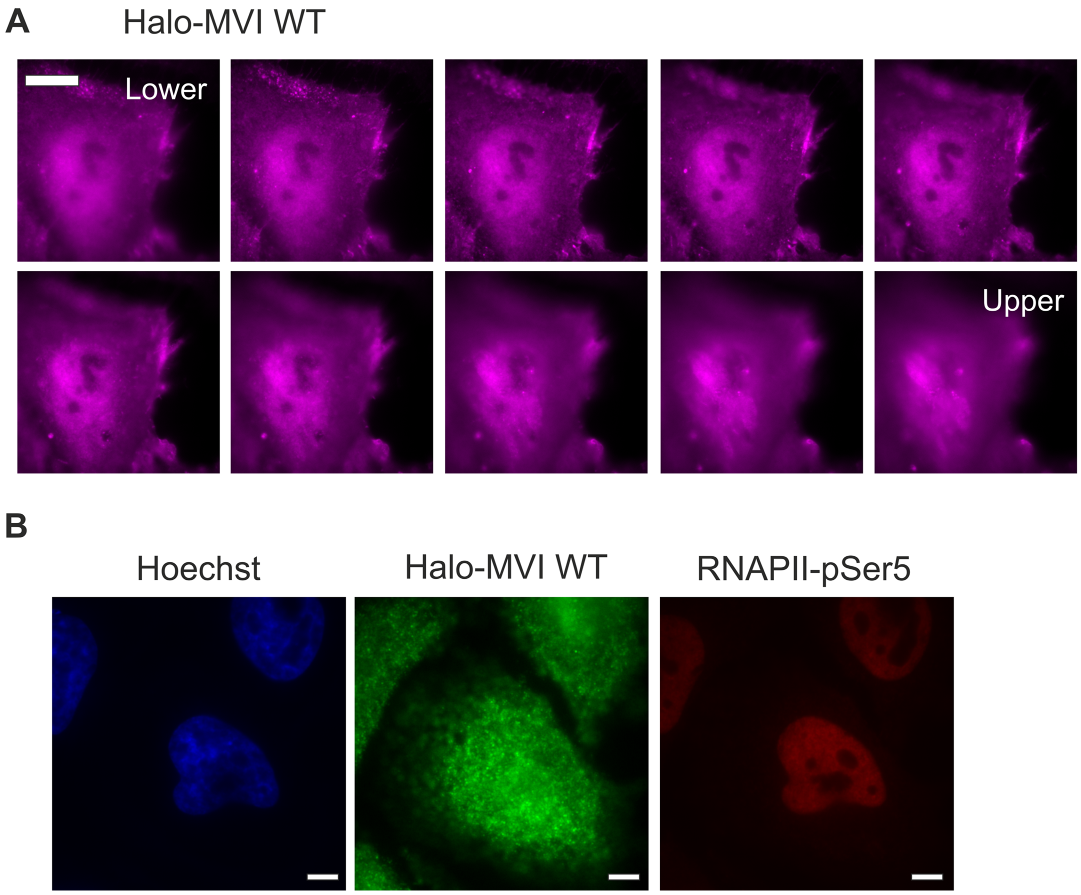

Supplementary Fig. 19 Nuclear localisation of Halo-MVI WT construct. (A) Halo-TMR staining against Halo-MVI in HeLa cells Images where acquired at 400 nm intervals in the z axis. (scale bar 10 μm). (B) Halo-TMR staining against Halo-MVI WT, DNA is shown in blue and immunofluorescence staining against RNAPII-pSer5 in HeLa cells (scale bar 5 μm). Z-stack is shown in Supplementary Movie 10.

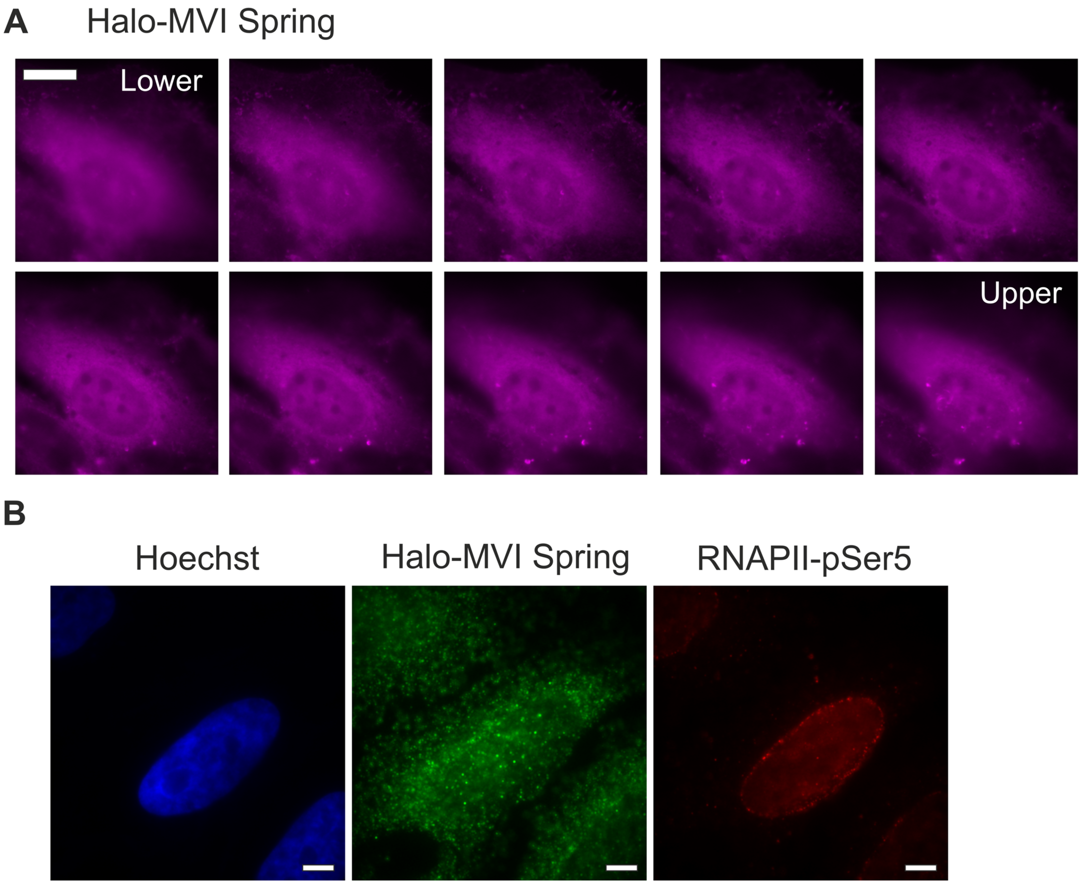

Supplementary Fig. 20 Nuclear localisation of Halo-MVI Spring construct. (A) Halo-TMR staining against Halo-MVI Spring in HeLa cells Images where acquired at 400 nm intervals in the z axis. (scale bar 10 μm). (B) Halo-TMR staining against Halo-MVI Spring, DNA is shown in blue and immunofluorescence staining against RNAPII-pSer5 in HeLa cells (scale bar 5 μm). Z-stack is shown in Supplementary Movie 11.

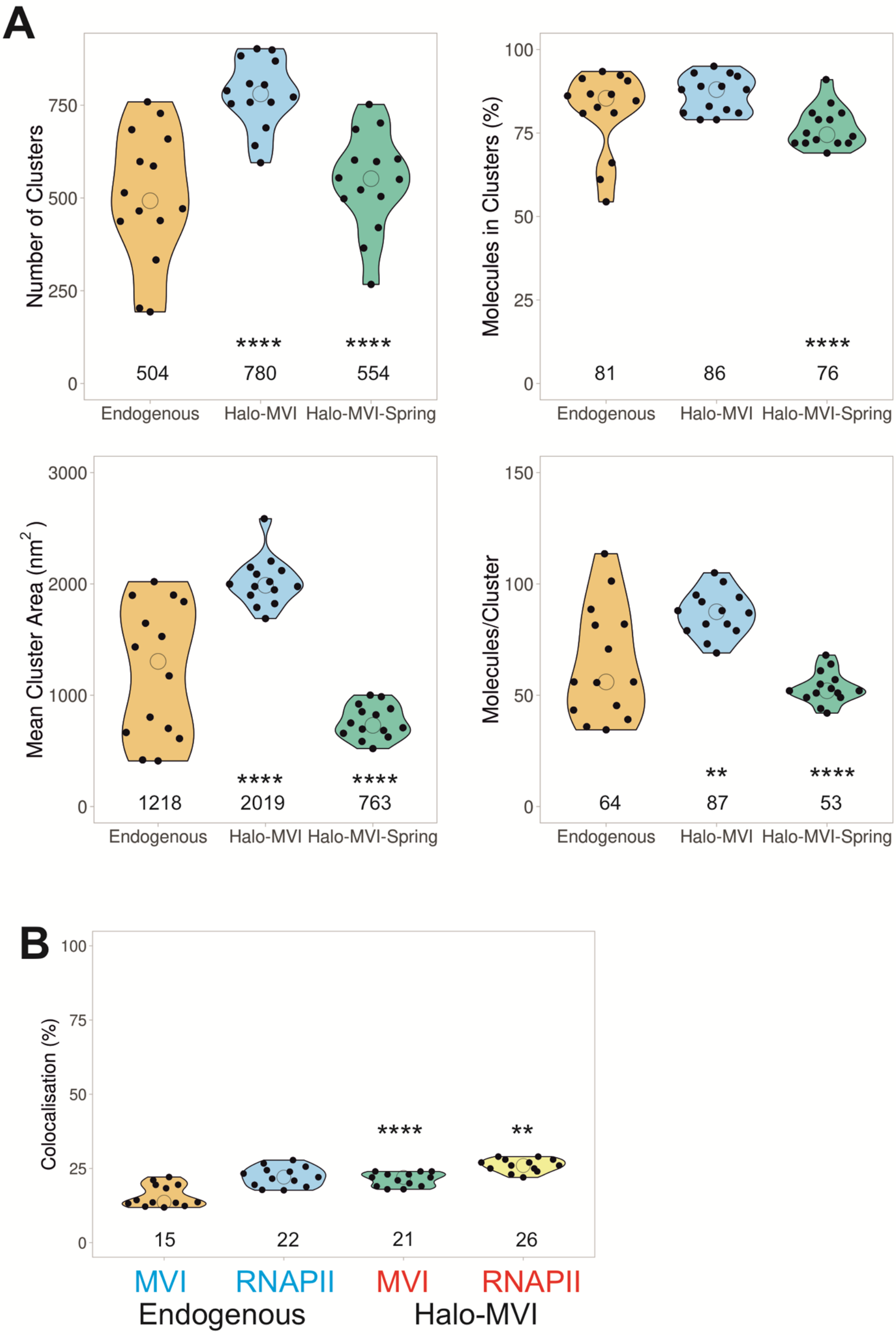

Supplementary Fig. 21 Nuclear localisation of Halo MVI constructs. (A) Cluster analysis of MVI nuclear organisation comparing endogenous protein (n=13), Over expression of Halo-WT-MVI (n=13) and Over expression of Halo-MVI-Spring (n=13). The values represent the mean from the ROIs for each condition (Only statistically significant changes are highlighted **p <0.01, ****p <0.0001 by two-tailed t-test). Halo-MVI was compared to endogenous and Halo-MVI-Spring was compared to Halo-MVI. (B) Colocalization analysis of myosin VI (MVI) and RNAPII-pSer5 clusters with endogenous protein (n=13) and over expression of Halo-WT-MVI (n=13). Individual data points represent the percentage of each protein which is colocalized based on a DoC value above 0.4. (Only statistically significant changes are highlighted ****p <0.0001 by two-tailed t-test compared to endogenous conditions for each protein).

Supplementary Table 1. GO Gene Expression analysis

False Discovery Rate (FDR) corrected p values were calculated from the hypergeometric test.

p value (FDR corrected)	Genes in List	Total Genes	Functional Category	GO Term
2.88E-09	308	3903	Regulation of cell communication	GO:0010646
2.88E-09	310	3952	Regulation of signaling	GO:0023051
7.76E-09	265	3287	Cell surface receptor signaling pathway	GO:0007166
7.76E-09	280	3529	Regulation of signal transduction	GO:0009966
7.20E-07	244	3113	Intracellular signal transduction	GO:0035556
1.71E-06	269	3547	Response to organic substance	GO:0010033
2.78E-06	169	2027	Regulation of intracellular signal transduction	GO:1902531
2.78E-06	204	2561	Response to external stimulus	GO:0009605
2.78E-06	218	2785	Anatomical structure morphogenesis	GO:0009653
2.78E-06	226	2905	Regulation of localization	GO:0032879
2.78E-06	151	1756	Regulation of cell proliferation	GO:0042127
2.78E-06	344	4820	Regulation of response to stimulus	GO:0048583
2.78E-06	256	3382	Regulation of multicellular organismal process	GO:0051239
2.78E-06	119	1286	Negative regulation of multicellular organismal process	GO:0051241
2.78E-06	64	551	Circulatory system process	GO:0003013
3.08E-06	63	542	Blood circulation	GO:0008015
3.85E-06	161	1921	Locomotion	GO:0040011
3.90E-06	197	2474	Nervous system development	GO:0007399
4.67E-06	99	1028	Regulation of cellular component movement	GO:0051270
9.19E-06	175	2165	Cell proliferation	GO:0008283

Supplementary Table 2. Recombinant DNA.

Construct	Source
Xenopus pFastBac1 Calmodulin (1-end)	Sellers Lab
Human pFastbacHTB MVI (1-1253)	Fili et al 2017
Human pFastbacHTB MVI bzip	Sellers Lab
Human pFastbacHTB MVI Spring	Synthetic Gene - This Study
Human pFastbacHTB MVI Spring bzip	Synthetic Gene - This Study
pHalo-Rpb1	Darzacq lab
pSNAP-C1	Addgene 58186
pSNAP-Rpb1	This Study
YFP-NLS-R62D Actin	de Lanerolle Lab
pLV-Tet0-Halo-NLS-Motor (1-814)	Große-Berkenbusch et al 2020
pLV-Tet0-Halo-NLS-CBD (1060-1253)	Große-Berkenbusch et al 2020
pcDNA3.1 MVI	Synthetic Gene - This Study
pcDNA3.1 MVI Spring	Synthetic Gene - This Study
pEGFP-NES-Motor (1-814)	Synthetic Gene - This Study
pEGFP-NES-CBD (1060-1253)	Synthetic Gene - This Study

Supplementary Table 3. Primers for qPCR.

Sequence	Use
CTGCTGCAGGAACCTCTCAT	VASP For
CTTCCTGGGGAGAATGTGG	VASP Rev
GCTACGGCAAAGTCTGCTCT	CDC42BPA Pro. For
TCACAACCACTGCAGTCTCC	CDC42BPA Pro. Rev
AGGGGGAATCTCTAGGCAAA	PTEN Pro. For
TGCATTCGCTCTTTCCTTTT	PTEN Pro. Rev
GAATGGTTTGTGGCTCAGGT	PTEN Int. For
CCCACTGGGTTGAAATATGG	PTEN Int. Rev
GGTGCCTGAGAAGAGGTGAG	EGR3 ex2 For
CCATGTGGATGAATGAGGTG	EGR3 ex2 Rev
TCACGTACCACACACACACG	EGR3 Int. For
TCCGGGTCTGAAACTACCTG	EGR3 Int. Rev
CCCCTGCTTCTTCTCAGTTG	IER3 Pro. For
AAAAGATGCACGGATTGGAG	IER3 Pro. Rev
CTCACCTTCCTCCTCCTCCT	TNNT1 Int. For
GTCCAGCAGAGACTGGAACC	TNNT1 Int. Rev
ATCAGGAGACAGAAGCCATAAAG	PTEN RT-qPCR For.
GTCTGTGTGTCTCGGCTAATC	PTEN RT-qPCR Rev.
GCGGCAAAGTAGGAGAAGAA	IER3 RT-qPCR For
GTAGACAGACGGAGTTGAGAATG	IER3 RT-qPCR Rev
GGAGAAGAACAGCACAACCT	VASP RT-qPCR For
CCCTCTGTAGGTCCGAGTAAT	VASP RT-qPCR Rev
TGGTCAAGGCAGAACAGAAG	TNNT1 RT-qPCR For
CCCATGTAGTCAATGTCCAGAG	TNNT1 RT-qPCR Rev
CTCGGATGGAGGTTACTCTTTC	INHA RT-qPCR For
CACCAGCCATGGGATTAAGA	INHA RT-qPCR Rev
TCAGCCCAGTGTTGTCATTAG	CDC42BPA RT-qPCR For
TCTTTCCGACTTGCATGGATAA	CDC42BPA RT-qPCR Rev
GGCTCCTTTGAAGTGGAGTAATA	EGR3 RT-qPCR For
CACAGGAGAAGTAACGCTAACA	EGR3 RT-qPCR Rev
GTCTCCTCTGACTTCAACAGCG	GAPDH RT-qPCR For
ACCACCCTGTTGCTGTAGCCAA	GAPDH RT-qPCR Rev
