# Supplementary material for: Myosin VI regulates the spatial organisation of mammalian transcription initiation
Source: Nat Commun. 2022 Mar 15;13:1346. doi: 10.1038/s41467-022-28962-w (PMC8924246; doi:10.1038/s41467-022-28962-w)
Supplement: Supplementary file 2 — Description for Additional Supplementary Files [file 41467_2022_28962_MOESM2_ESM.docx]

**Supplementary Movie 1**

Widefield Immunofluorescence staining against MVI (magenta) and DNA (cyan) in HeLa cells under Normal growth conditions, as shown in Figure 1B.

**Supplementary Movie 2**

Representative movie of J549 staining against Halo-RNAPII in live HeLa cells, as shown in Figure 5.

**Supplementary Movie 3**

Representative movie of J549 staining against Halo-RNAPII in live HeLa cells in the presence of TIP, as shown in Figure 5.

**Supplementary Movie 4**

Widefield Immunofluorescence staining against MVI (magenta) and DNA (cyan) in HeLa cells following Serum Stimulation, as shown in Supplementary Figure 3.

**Supplementary Movie 5**

Widefield Immunofluorescence staining against MVI (magenta) and DNA (cyan) in HeLa cells following Serum starvation, as shown in Supplementary Figure 4.

**Supplementary Movie 6**

Immunofluorescence staining of Myosin VI and RNAPII-pSer5 following TIP-treatment, as shown in Supplementary Figure 7.

**Supplementary Movie 7**

Immunofluorescence staining of RNAPII-pSer5 following MVI siRNA knockdown, as shown in Supplementary Figure 10.

**Supplementary Movie 8**

Halo-TMR and Immunofluorescence staining against NLS myosin VI motor (red) and RNAPII-pSer5 (green) in HeLa cells, as shown in Supplementary Figure 12.

**Supplementary Movie 9**

Halo-TMR and Immunofluorescence staining against NLS myosin VI CBD (red) and RNAPII-pSer5 (green) in HeLa cells, as shown in Supplementary Figure 12.

**Supplementary Movie 10**

Halo-TMR staining against Halo-MVI WT, DNA is shown in blue and immunofluorescence staining against RNAPII-pSer5 in HeLa cells, as shown in Supplementary Figure 19.

**Supplementary Movie 11**

Halo-TMR staining against Halo-MVI Spring, DNA is shown in blue and immunofluorescence staining against RNAPII-pSer5 in HeLa cells, as shown in Supplementary Figure 20.
