# Supplementary material for: Myosin VI regulates the spatial organisation of mammalian transcription initiation
Source: Nat Commun. 2022 Mar 15;13:1346. doi: 10.1038/s41467-022-28962-w (PMC8924246; doi:10.1038/s41467-022-28962-w)
Supplement: Supplementary file 14 — Reporting Summary [file 41467_2022_28962_MOESM14_ESM.pdf]

nature research

Corresponding author(s): Christopher P. Toseland

Last updated by author(s): Dec 16, 2020

Reporting Summary

Nature Research wishes to improve the reproducibility of the work that we publish. This form provides structure for consistency and transparency in reporting. For further information on Nature Research policies, see our Editorial Policies and the Editorial Policy Checklist.

Statistics

For all statistical analyses, confirm that the following items are present in the figure legend, table legend, main text, or Methods section.

n/a	Confirmed	
☐	☒	The exact sample size (*n*) for each experimental group/condition, given as a discrete number and unit of measurement
☐	☒	A statement on whether measurements were taken from distinct samples or whether the same sample was measured repeatedly
☐	☒	The statistical test(s) used AND whether they are one- or two-sided<br>*Only common tests should be described solely by name; describe more complex techniques in the Methods section.*
☒	☐	A description of all covariates tested
☒	☐	A description of any assumptions or corrections, such as tests of normality and adjustment for multiple comparisons
☐	☒	A full description of the statistical parameters including central tendency (e.g. means) or other basic estimates (e.g. regression coefficient) AND variation (e.g. standard deviation) or associated estimates of uncertainty (e.g. confidence intervals)
☐	☒	For null hypothesis testing, the test statistic (e.g. *F*, *t*, *r*) with confidence intervals, effect sizes, degrees of freedom and *P* value noted<br>*Give P values as exact values whenever suitable.*
☒	☐	For Bayesian analysis, information on the choice of priors and Markov chain Monte Carlo settings
☒	☐	For hierarchical and complex designs, identification of the appropriate level for tests and full reporting of outcomes
☒	☐	Estimates of effect sizes (e.g. Cohen's *d*, Pearson's *r*), indicating how they were calculated

Our web collection on statistics for biologists contains articles on many of the points above.

Software and code

Policy information about availability of computer code

Data collection	Commercial microscopy software (Zeiss ZEN Black) was used to acquire STORM and confocal data. Nikon instruments (NIS-Elements) software was used to gather single molecule data. Biochemical Data was collected using commercial BMG-Labtech software running Clariostar plate reader system.
Data analysis	Zeiss ZEN Black was used to process the STORM data. All images were exported as TIFFs. STORM localisation tables were exported as text files for input in to open software (Clus-DoC). Single molecule tracking data was acquired in nd2 format and then exported as TIFFs. Tracking files are saved as text files. ImageJ (V1.53K) was used to produce final images.

For manuscripts utilizing custom algorithms or software that are central to the research but not yet described in published literature, software must be made available to editors and reviewers. We strongly encourage code deposition in a community repository (e.g. GitHub). See the Nature Research guidelines for submitting code & software for further information.

Data

Policy information about availability of data

All manuscripts must include a data availability statement. This statement should provide the following information, where applicable:

- Accession codes, unique identifiers, or web links for publicly available datasets
- A list of figures that have associated raw data
- A description of any restrictions on data availability

All Raw data is available upon request to the Corresponding author and Source data is provided in the Source Data file. RNA-Seq data were deposited in the Gene Expression Omnibus (GEO) database under the accession number GSE149448

Field-specific reporting

Please select the one below that is the best fit for your research. If you are not sure, read the appropriate sections before making your selection.

- [x] Life sciences
- [ ] Behavioural & social sciences
- [ ] Ecological, evolutionary & environmental sciences

For a reference copy of the document with all sections, see nature.com/documents/nr-reporting-summary-flat.pdf

Life sciences study design

All studies must disclose on these points even when the disclosure is negative.

Sample size	Besides microscopy, all experiments were performed in triplicate or greater. STORM images were performed on a minimum of 10 cells per condition. Single molecule tracking experiments were performed on a minimum of 100 cells per condition. The microscopy numbers were determined prior to experiment based on timing/scale of the specific experiment. Post-determination was then applied by measuring statistic differences between the treatments where more experiments would be performed if variance prevented data determination.
Data exclusions	None
Replication	Imaging experiments were repeated on multiple occasions (at least 4 times). RNA-seq experiments were performed in triplicate but not repeated independently due to costs. All other experiments were repeated in triplicate. All replicates were successful.
Randomization	Conditions were grouped based on treatments and experiment group.
Blinding	Imaging experiments were not blinded because phenotypic changes were clear (e.g. lack of protein due to knockdown). Other experiments were blinded.

Reporting for specific materials, systems and methods

We require information from authors about some types of materials, experimental systems and methods used in many studies. Here, indicate whether each material, system or method listed is relevant to your study. If you are not sure if a list item applies to your research, read the appropriate section before selecting a response.

Materials & experimental systems

n/a	Involved in the study	
☐	☒	Antibodies
☐	☒	Eukaryotic cell lines
☒	☐	Palaeontology and archaeology
☒	☐	Animals and other organisms
☒	☐	Human research participants
☒	☐	Clinical data
☒	☐	Dual use research of concern

Methods

n/a	Involved in the study	
☒	☐	ChIP-seq
☒	☐	Flow cytometry
☒	☐	MRI-based neuroimaging

Antibodies

Antibodies used	Rabbit anti-myosin VI (Atlas-Sigma HPA0354863), Rabbit anti-Histone H3 (tri methyl K9) (Abcam ab8898), Rabbit anti-Histone H3 (acetyl K27) (Abcam ab4729), Rabbit anti-Histone H3 (acetyl K9) (Abcam ab4441), Rabbit anti-RNAPII phospho Ser5 (Abcam Ab5131), Mouse anti-RNAPII phospho Ser5 (Abcam Ab5408), Donkey anti-mouse Alexa Fluor 488-conjugated (Abcam Ab181289), Donkey anti-rabbit Alexa Fluor 647-conjugated (Abcam Ab181347) and Donkey anti-rabbit Alexa Fluor 488-conjugated antibody (Abcam Ab181346).
Validation	Target Validation has been performed by the suppliers: ab8898, ab4729, ab4441, ab5131, ab5408 through blocking of antibody by immunizing peptide against specific modifications. Secondary antibodies were validated for non-specific interactions by performing experiments in the absence of primary antibody.<br><br>. Myosin VI antibody validation is also confirmed by siRNA knockdown of endogeneous myosin VI in this paper.

Eukaryotic cell lines

Policy information about cell lines

Cell line source(s)	HeLa (ECACC 93021013), Spodoptera frugiperda Sf9 (ThermoFisher 11496015) and Sf21 (ThermoFisher 11497013) cells
Authentication	Authenticated by supplier: HeLa through STR-PCR and Karyotype. sf9 and sf21 through morphology.

Mycoplasma contamination	Not detected upon testing.
Commonly misidentified lines (See ICLAC register)	No commonly misidentified cell lines were used in this study.
